# Is there a bidirectional relationship between the number of unhealthy lifestyle factors and depressive symptoms in adolescents? Evidence from a longitudinal study

**DOI:** 10.1186/s12916-026-04984-9

**Published:** 2026-06-10

**Authors:** Meiqi Wang, Afei Qin, Suyun Li, Cuixia Lv, Zhaolu Liu, Shoujuan Zheng, Lianlong Yu, Jie Zhang

**Affiliations:** 1 Department of Social Medicine and Health Management, School of Public Health, Cheeloo College of Medicine, Shandong University, Jinan, 250012 China; 2 NHC Key Lab of Health Economics and Policy Research (Shandong University), Jinan, 250012, China; 3 Center for Health Management and Policy Research, Shandong University (Shandong Provincial Key New Think Tank), Jinan, 250012, China; 4 School (Institute) of Mental Health and Psychological Sciences, Cheeloo College of Medicine, Shandong University, Jinan, 250012 China; 5 Shandong Center for Disease Control and Prevention, Jinan, 250014, China; 6 School of Public Health, Cheeloo College of Medicine, Shandong University, Jinan, 250012 China; 7 School of Public Health, Shandong First Medical University & Shandong Academy of Medical Sciences, Jinan, 250117 China; 8 School of Public Health, Shandong Second Medical University, Weifang, 261053 China; 9 Department of Sociology, SUNY Buffalo State University, Buffalo, NY 14222 USA

**Keywords:** Adolescents, Depression, Unhealthy lifestyle behaviors, Longitudinal analysis

## Abstract

**Background:**

Depression and unhealthy lifestyle factors are major public health concerns. Although individual unhealthy lifestyle factors have been associated with adolescent depressive symptoms, the longitudinal relationships between depressive symptoms, the cumulative number of unhealthy lifestyle factors, and depressive symptom trajectories remain unclear. This study examined these associations among Chinese adolescents.

**Methods:**

Three annual waves of longitudinal data (T1: 2022; T2: 2023; T3: 2024) were collected using self-administered paper-based questionnaires and multi-stage stratified cluster sampling. The study included 5,988 adolescents (mean baseline age, 12.75 years; 3,013 boys [50.3%]). Depressive symptoms were assessed using the 20-item Center for Epidemiologic Studies Depression Scale. Eight unhealthy lifestyle factors were assessed: smoking, alcohol consumption, physical inactivity, low fruit/vegetable intake, unhealthy diet, excessive screen time, sedentary behavior, and inadequate sleep. Random intercept cross-lagged panel models examined bidirectional associations between depressive symptoms and the number of unhealthy lifestyle factors, adjusting for age, gender, ethnicity, region, living with father/mother, living on campus, education level, and body mass index. Latent class linear mixed models identified depressive symptom trajectories, and generalized linear mixed models examined associations between these trajectories and the number of unhealthy lifestyle factors.

**Results:**

Depressive symptoms at time T significantly predicted the number of unhealthy lifestyle factors at time T + 1, whereas the reverse associations were not significant. Three depressive symptom trajectories were identified: “low” (*n* = 4,401), “fluctuating” (*n* = 1,264), and “high peaking” (*n* = 323). Compared with the “low” group, adolescents in the “fluctuating” and “high peaking” groups had a significantly higher number of unhealthy lifestyle factors at T3 (AIRR = 1.128, 95% CI: 1.088–1.171; AIRR = 1.162, 95% CI: 1.092–1.236). However, the number of unhealthy lifestyle factors at T1 was not significantly associated with later depressive symptoms or depressive symptom trajectories. Sensitivity analyses supported this unidirectional temporal pattern.

**Conclusions:**

Depressive symptoms may precede the accumulation of multiple unhealthy lifestyle factors in adolescents, whereas the reverse direction was not supported. Adolescents with “fluctuating” or “high peaking” depressive symptom trajectories showed greater behavioral risk accumulation. Long-term monitoring and early intervention for depressive symptoms may help prevent recurrent symptoms and unhealthy lifestyle consequences.

**Supplementary Information:**

The online version contains supplementary material available at 10.1186/s12916-026-04984-9.

## Background

Depression and unhealthy lifestyle behaviors are both critical global public health issues. Adolescence is a critical stage for the onset and development of depression [1]. Adolescent-onset depression is recurrent and leads to more severe and persistent psychosocial impairments compared with adult-onset depression [2]. Approximately one in five children and adolescents worldwide experience depression or depressive symptoms [3]. In China, the pooled prevalence of depressive symptoms among children and adolescents is 26.17% [4]. According to the World Health Organization (WHO), depression arises from a complex interplay of social, psychological, and biological factors [5]. Unhealthy lifestyle behaviors (e.g., smoking, alcohol consumption, physical inactivity, low fruit and vegetable intake, poor diet quality, excessive screen time, sedentary behavior, and insufficient sleep duration) have been widely recognized as risk factors for depression [6].

Numerous studies have examined the relationships between various unhealthy lifestyle factors and depressive symptoms in adolescents. A body of longitudinal research indicates a significant bidirectional association between smoking and depression, while two reviews suggest that the causal relationship between smoking and depression remains uncertain in adolescents [7–9]. One prospective cohort study has found a positive association between alcohol consumption at age 18 and depression at age 24 [10]. During adolescence, both weekly alcohol use and heavy drinking, even in the absence of an alcohol use disorder (AUD), can increase the risk of depression [11]. Regarding physical inactivity, a recent birth cohort study demonstrated that more severe depressive symptoms in adolescents aged 10–12 and 14–16 were associated with shorter subsequent durations of daily physical activity, including moderate-to-vigorous physical activity (MVPA) [12]. Furthermore, higher levels of physical activity at ages 14–16 and 16–19 were linked to milder depressive symptoms later on [12]. Other studies have also reported an inverse relationship between physical activity and depressive symptoms in adolescents [13, 14]. A meta-analysis concluded that fruit and vegetable intake is significantly associated with a lower risk of depression in adolescents [15]. Other reviews have reported that unhealthy and poor-quality diets are related to depression and depressive symptoms in children and adolescents [16, 17]. A cohort study of 3,826 adolescents found that increased screen time exacerbates depressive symptoms [18]. Another longitudinal study involving 3,088 adolescents found that adolescents who developed smartphone dependence were at higher risk of depressive symptoms [19]. Adolescents with increasing sedentary behavior tend to experience depressive symptoms and have higher depressive symptom scores [20, 21]. A systematic review showed that both longitudinal and cross-sectional studies have identified associations between insufficient sleep and the severity of depressive symptoms [22]. Evidence from the literature suggests that insufficient sleep significantly increases the risk of depression in adolescents by impairing mood valence and emotion regulation [23].

Beyond investigating the independent relationships between individual unhealthy lifestyle factors and depression, a growing number of studies have focused on the clustering of unhealthy lifestyle behaviors [24–26]. A recent cross-sectional study using the National Health and Nutrition Examination Survey (NHANES) database investigated the cumulative effects of unhealthy lifestyle behaviors on depression among US adults [27]. This study found that the risk of depression increases with a greater number of unhealthy lifestyle behaviors [27]. Among adolescents, a cross-sectional study identified a dose-response relationship between the number of unhealthy lifestyle factors and depressive symptoms [28]. A systematic review and meta-analysis also found that unhealthy lifestyle factors collectively contribute to depressive symptoms [29]. Although different unhealthy lifestyle factors have been established to be associated with depressive symptoms in adolescents, most existing studies have employed cross-sectional designs, leaving the longitudinal relationship between the number of unhealthy lifestyle factors and depressive symptoms unclear.

Given the unclear longitudinal relationship described above, it is necessary to evaluate the longitudinal dose-response association between depressive symptoms and the subsequent number of unhealthy lifestyle behaviors. This analysis allows us to characterize their dynamic relationship and to identify symptom ranges in which the risk of accumulating multiple unhealthy lifestyle behaviors changes most markedly.

Trajectory analysis has become increasingly common and has substantial value for research on the development of depression. A growing body of longitudinal studies has identified distinct developmental trajectories of depressive symptoms in adolescents, which may reflect different underlying psychosocial and neurodevelopmental mechanisms [30]. However, the relationship between different types of depressive symptom trajectories and the number of unhealthy lifestyle behaviors remains unclear. Only one recent longitudinal study has reported the associations between depressive symptom trajectories and different unhealthy lifestyle factors [31], yet it did not account for the number of unhealthy lifestyle behaviors.

This study focuses on depressive symptoms rather than clinically diagnosed depression, as depressive symptoms represent a persistent and subclinical state of emotional distress. Compared with clinically diagnosed depression, depressive symptoms are more common among adolescents [3]. To address the above gaps, this study aims to: (1) examine the longitudinal relationship between the number of unhealthy lifestyle factors and depressive symptoms, taking into account both within-person and between-person components; (2) investigate whether a non-linear relationship exists between the number of unhealthy lifestyle factors and depressive symptoms; (3) identify characteristic profiles of depressive symptom trajectories; and (4) explore the association between depressive symptom trajectories and the number of unhealthy lifestyle factors. This research will help advance understanding of the longitudinal relationship between depressive symptoms and the accumulation of unhealthy lifestyle factors. These insights may help inform integrated interventions that simultaneously address depressive symptoms and unhealthy lifestyle factors.

## Methods

### Study design and participants

This study used three waves of data (T1: 2022; T2: 2023; T3: 2024) from the Surveillance of Students’ Common Diseases and Health Influencing Factors (SSCDHIF), an annual survey conducted in Shandong Province, China. The SSCDHIF aims to monitor the prevalence of common diseases and associated health-related factors among students, thereby strengthening the prevention and control of student health issues and promoting the well-being of children and adolescents. Data were collected via a multi-stage stratified cluster sampling design. In each urban district of Shandong Province, two junior high schools, two senior high schools, and one vocational high school were randomly selected, whereas in each county, two junior high schools and one senior high school were selected. Within each selected school, two or more classes were randomly chosen from each grade. The schools participating in the surveillance program were intended to remain consistent across annual waves. All students in the selected classes were included in the survey, resulting in at least 80 participants per grade. Overall, a minimum of 240 students were sampled from each high school. Paper-based questionnaires were distributed to students after written informed consent was obtained. In accordance with the national monitoring program, this monitoring project was reviewed and approved by the National Expert Committee [32]. The study protocol was also approved by the Medical Ethics Committee of the Shandong Center for Disease Control and Prevention. Trained investigators delivered the questionnaires to participating schools, where teachers assisted with their distribution and collection. Investigators then reviewed all returned questionnaires, and any with incomplete or inconsistent responses were followed up for clarification or completion.

The sample comprised middle school students from junior high schools and senior high schools, including vocational high schools. The numbers of schools sampled and participating in the study were 788 in T1, 806 in T2, and 807 in T3, respectively. All students in the selected classes participated in the survey. The numbers of participants for the three years were 147,011 at T1, 154,477 at T2, and 156,281 at T3, respectively (Additional file 1: Figure S1). After linking data across all three waves, a total of 6,165 Chinese adolescents were included in the final longitudinal sample. The SSCDHIF survey was designed as a multi-wave repeated cross-sectional survey using school-class sampling. Therefore, the probability of students being included in subsequent survey waves depended on whether their grade level remained within the sampling frame at the next time point. As shown in Additional file 1: Table S1, the attrition analysis indicated that older students (i.e., those at higher education levels) had significantly higher attrition rates in later waves. This was entirely consistent with the sampling design, as students in higher grades naturally exited the sampling frame as they graduated or advanced to grades not included in the survey sampling frame. Thus, the longitudinal sample that participated in all three waves (*n* = 6,165) represented a subset naturally formed from the original cross-sectional sample due to the sampling design. Due to missing data, 5,988 participants were ultimately included in this study.

### Depressive symptoms

The 20-item Center for Epidemiologic Studies Depression Scale (CES-D) [33] was used to assess depressive symptoms in adolescents over the preceding week. This scale has demonstrated good reliability and validity in Chinese student populations [34]. Each item is rated on a 4-point Likert-type scale, ranging from 0 (rarely or none of the time [less than 1 day]) to 3 (most or all of the time [5–7 days]). Items 4, 8, 12, and 16 are reverse scored. Total scores range from 0 to 60, with higher scores reflecting more severe depressive symptoms. The scale demonstrated good internal consistency at the three time points (T1, T2, and T3), with Cronbach’s α values of 0.845, 0.870, and 0.869, respectively.

### Number of unhealthy lifestyle factors

In this study, unhealthy lifestyle factors included smoking, alcohol consumption, physical inactivity, low fruit/vegetable intake, unhealthy diet, excessive screen time, sedentary behavior, and inadequate sleep. The unhealthy lifestyle factors included were selected based on their established association with mental health outcomes in adolescents, their inclusion in the national health monitoring program for primary and middle school students in China, and their consistent availability across survey waves. Other behaviors were not included because they were either not assessed in the current monitoring system or exhibited low prevalence in the study population. To assess the validity of these eight unhealthy lifestyle factors, a confirmatory factor analysis was conducted. The evaluated fit indices were as follows: Goodness-of-Fit Index (GFI) = 0.998, Adjusted Goodness-of-Fit Index (AGFI) = 0.995, Comparative Fit Index (CFI) = 0.958, Tucker–Lewis Index (TLI) = 0.927, Root Mean Square Error of Approximation (RMSEA) = 0.022, and Standardized Root Mean Square Residual (SRMR) = 0.014. The results indicated good model fit, supporting the validity of the construct.

Smoking (specifically tobacco smoking, excluding vaping/e-cigarettes) and alcohol consumption were assessed using the following questions: “Have you ever smoked, even one or two puffs?” and “Have you ever drunk a full glass of alcohol?”, respectively. Responses were coded as yes (1) or no (0). Physical inactivity was measured by the question, “In the past 7 days, how many days did you engage in at least 60 minutes of moderate-to-vigorous physical activity per day?”, with responses ranging from 0 to 7 days. According to WHO and Chinese guidelines, participants who reported 0–6 days of such activity were classified as physically inactive (1), whereas those reporting activity on all 7 days were classified as active (0) [35, 36]. Low fruit/vegetable intake was assessed with two questions: “In the past 7 days, how many times did you eat fresh fruit?” and “In the past 7 days, how many times did you eat vegetables?” Response options included “never,” “less than once a day,” “once a day,” and “twice a day or more.” Based on Chinese guidelines, participants who consumed both fruits and vegetables at least once daily were coded as 0; those who did not meet this criterion for either food group were coded as 1 [36]. Unhealthy diet was assessed using two questions: “In the past 7 days, how many times did you drink sugar-sweetened beverages?” and “In the past 7 days, how many times did you eat fried food?” Response options were “never,” “less than once a day,” and “once a day or more.” Based on Chinese guidelines and previous studies, participants reporting consumption of either sugar-sweetened beverages or fried food at least once per day were coded as 1; all others were coded as 0 [36, 37]. Screen time was defined as the average daily time spent watching TV, using computers, or using mobile electronic devices in the past week. Based on Chinese guidelines and previous studies, daily screen time exceeding 2 h was coded as 1 (excessive), while 2 h or less was coded as 0 [38, 39]. Sedentary behavior was assessed using the question, “In the past week, what was your average daily sedentary time indoors?” Based on previous studies, daily sedentary time exceeding 2 h was coded as 1, while 2 h or less was coded as 0 [40]. Based on Chinese guidelines, inadequate sleep was defined differently by education level: for junior high school students, less than 9 h of sleep per night; for senior high school students, less than 8 h per night [41]. For a sensitivity analysis, the continuous number of unhealthy lifestyle factors (range: 0–8) was dichotomized into two categories: 0–3 and 4–8 factors.

### Covariates

This analysis included both time-invariant covariates—age, gender (boys = 1, girls = 0), ethnicity (Han = 1, other = 0), region (urban = 1, suburban = 0), living with father/mother (yes = 1, no = 0), and living on campus (yes = 1, no = 0)—and time-varying covariates, including education level (senior high school = 1, junior high school = 0) and body mass index (BMI). Although region, living with father/mother, and living on campus showed minor differences in their distributions across waves, the between-wave changes were negligible, indicating high temporal stability. Therefore, they were treated as time-invariant rather than time-varying covariates. Age was calculated based on the year of birth at each survey wave. Region referred to the location of the school. Living with father/mother was assessed using the question, “In the past six months, which family members have lived with you?” A response including “father” or “mother” was coded as “yes”; otherwise, it was coded as “no.” Living on campus was assessed by the question “Do you live on campus?” with responses “yes” or “no.” BMI was computed as weight in kilograms divided by the square of height in meters (kg/m^2^). Age and BMI were treated as continuous variables and were standardized (z-scored) for analysis, while all other covariates were dichotomous.

### Statistical analysis

The analytical sample comprised participants who completed questionnaires at all three time points (T1, T2, and T3). Missing data were handled using listwise deletion, as the proportion of missing values was less than 5%. Descriptive analyses presented means and standard deviations (SDs) for continuous variables and frequencies and percentages for categorical variables at T1, T2, and T3. Differences in proportions of the same variables between two time points were examined using McNemar’s test. Differences in mean values of the same variables between two time points were analyzed using paired t-tests or Wilcoxon signed-rank tests, as appropriate. Spearman correlation analysis was used to explore the correlation between depressive symptoms and the number of unhealthy lifestyle factors at T1, T2, and T3.

The random intercept cross-lagged panel model (RI-CLPM) was employed to examine the longitudinal associations between depressive symptoms and the number of unhealthy lifestyle factors across the three time points (T1, T2, and T3), which allows for the decomposition of observed variance into stable between-person components and dynamic within-person fluctuations [42, 43]. Model parameters were estimated using the maximum likelihood estimator with robust standard errors (MLR) to account for non-normality in the data. Six RI-CLPMs were estimated sequentially in this study: Model a (baseline model); Model b (Model a with time-invariant covariates: age, gender, ethnicity, region, living with father/mother, and living on campus at T1); Model c (Model b with time-varying covariates: education level and BMI at T1, T2, and T3); Model d (Model c with autoregressive paths constrained to be equal across time); Model e (Model c with cross-lagged paths constrained to be equal across time); Model f (Model c with both autoregressive and cross-lagged paths constrained to be equal across time). Model comparisons were conducted exclusively for Models c–f, as these models adjusted for the same covariates [44]. Model fit was evaluated using the chi-square to degrees of freedom ratio (*χ*^2^/*df*), CFI, TLI, RMSEA, and SRMR. Good fit was indicated by CFI and TLI > 0.90, RMSEA and SRMR < 0.08 [45]. Smaller values of the Akaike Information Criterion (AIC) and Bayesian Information Criterion (BIC) also indicated better model performance. To achieve parsimony, a series of models with constrained parameters (Models d–f) were compared to the unconstrained model (Model c) using the Satorra-Bentler scaled chi-square difference test (S–Bχ²) [46, 47]. If the test was non-significant, the more parsimonious constrained model was retained. Standardized path coefficients were interpreted as small (0.03), medium (0.07), or large (0.12) [48, 49].

Because the CES-D scale included items related to sleep (Item 11) and physical activity (Items 7 and 20), there were potential risks that the observed associations between depressive symptoms and the number of unhealthy lifestyle behaviors might be driven, at least in part, by conceptual overlap between the measures. To minimize bias arising from such behavioral overlap, six additional sensitivity analyses were conducted based on Model c, controlling for covariates. Specifically, (1) Model g excluded inadequate sleep from the number of unhealthy lifestyle factors; (2) Model h excluded the sleep-related item from the depressive symptom score (Item 11: “My sleep was restless”); (3) Model i excluded sleep-related components from both the number of unhealthy lifestyle factors and the depressive symptom score; (4) Model j excluded physical inactivity from the number of unhealthy lifestyle factors; (5) Model k excluded physical inactivity-related items from the depressive symptom score (Item 7: “I felt that everything I did was an effort,” and Item 20: “I could not get ‘going’”); and (6) Model l excluded physical inactivity-related components from both the number of unhealthy lifestyle factors and the depressive symptom score. Finally, given that the RI-CLPM handles missing data under the full information maximum likelihood (FIML), an additional analysis was conducted including all 6,163 participants with available data (two participants were excluded from the initial sample of 6,165 due to missing data on the living with father/mother variable). This model additionally adjusted for both time-invariant and time-varying covariates (Model m).

A restricted cubic spline (RCS) model implemented using the “rms” package was used to explore the potential non-linear relationship between depressive symptoms in T1 and the number of unhealthy lifestyle factors in T3. Specifically, the number of unhealthy lifestyle factors at T3 was treated as the dependent variable, and depressive symptoms at T1 as the independent variable. The RCS model was adjusted for the following variables at T1: age (z-score), gender, ethnicity, region, living with father/mother, living on campus, education level, BMI (z-score), and the number of unhealthy lifestyle factors. The RCS model was fitted with 3 to 5 knots placed across the distribution of depressive symptoms at T1. The optimal number of knots was determined by comparing the AIC values across models with different knot numbers, and the model with the smallest AIC was selected as the final model. The model was estimated using ordinary least squares regression, and the statistical significance of the non-linear term was assessed using an F-test.

To examine the relationship between trajectories of depressive symptoms and unhealthy lifestyle factors, a latent class linear mixed model (LCMM) was conducted using the “lcmm” package to identify distinct trajectories of depressive symptoms over age. Specifically, we used the total CES-D scale score assessed at all three time points (T1, T2, and T3) as the longitudinal outcome. Age at each assessment served as the time variable, allowing us to model changes in depressive symptoms across adolescence. Several models specifying between one and four trajectory classes were tested. Model selection was guided by the Average Posterior Probability of Assignment (APPA > 0.70), Odds of Correct Classification (OCC > 5.00), relative entropy (higher values preferred), BIC (lower values preferred), and ensuring a minimum class proportion of ≥ 5% [50]. If no model met all criteria, the model with the lowest BIC was selected [38]. The depressive symptom trajectory classes were used as a categorical variable in subsequent models. Univariate analyses were performed to examine the differences in demographic variables and unhealthy lifestyle factors at the three time points (T1, T2, and T3) across depressive symptom trajectories. The Wilcoxon rank-sum test was used to examine differences in CES-D scores both between participants of different ages within the same trajectory group and between participants from different trajectory groups at the same age.

To account for potential clustering of student responses within schools and to control for unmeasured school-level heterogeneity, generalized linear mixed models (GLMMs) using the “lme4” and “lmerTest” packages with the school included as a random intercept were used to investigate the association between depressive symptom trajectories and the number of unhealthy lifestyle factors at T3, controlling for covariates at T1. Given the non-normal distribution, a Poisson GLMM was initially considered. The “DHARMa” package was used to test for overdispersion. If overdispersion was absent, the Poisson model was retained. If overdispersion was present, a negative binomial model was fitted, and the Vuong test was employed to compare it against a zero-inflated negative binomial model to select the better-fitting model. The results were reported as adjusted incidence rate ratios (AIRRs) with 95% confidence intervals (CIs). To assess the robustness of our primary findings, sensitivity analyses were performed to examine whether the longitudinal association between depressive symptoms and the number of unhealthy lifestyle factors remained consistent under different methodological assumptions. Specifically, three sensitivity analyses were conducted: (1) Model S1: exploring the association between depressive symptoms at T1 and the number of unhealthy lifestyle factors at T3 using a Poisson GLMM [AIRR (95% CI)]; (2) Model S2: exploring the association between depressive symptom trajectories and the number of unhealthy lifestyle factors at T3 using linear mixed models (LMMs) [β (95% CI)]; (3) Model S3: exploring the association between depressive symptom trajectories and dichotomized unhealthy lifestyle factors at T3 using a GLMM with a binomial distribution [adjusted odds ratios (AORs) (95% CI)].

The same GLMM analytical approach (Poisson/negative binomial) was applied to examine the association between the number of unhealthy lifestyle factors at T1 and depressive symptoms at T3, reporting AIRR (95% CI). A multinomial GLMM was used to assess the association between the number of unhealthy lifestyle factors at T1 and depressive symptom trajectory classes, reporting AORs (95% CIs). For all pairwise comparisons in GLMMs, p-values were adjusted using the Benjamini-Hochberg procedure to control the false discovery rate (FDR) via the “emmeans” package. For all statistical analyses except post-hoc comparisons, a two-sided p-value < 0.05 was considered statistically significant. Statistical analyses were performed using R version 4.5.1 and Mplus version 8.3. The primary statistical analyses were performed in R, including descriptive analyses, Spearman correlation analyses, RCS models, LCMMs, and GLMMs. The RI-CLPMs were estimated using Mplus.

## Results

### Descriptive statistics

Demographic characteristics of the participants at T1, T2, and T3 are presented in Table 1. Among the 5,988 adolescent participants with a mean baseline age of 12.75 years, 3,013 (50.3%) were boys and 2,975 (49.7%) were girls. At baseline, the majority of participants (85.1%) were junior high school students. Most participants lived with at least one parent (97.4%), and 22.0% reported living on campus. At T2 and T3, participants were correspondingly older, with mean ages of 13.75 and 14.75 years, respectively. The proportion of senior high school students increased over time, reaching 17.4% at T2 (*p* < 0.001) and 23.0% at T3 (*p* < 0.001).

**Table 1:** Demographic characteristics of the study participants at T1, T2, and T3

Variables	Frequency (%) or mean (SD)		
	T1	T2	T3
Total	5,988 (100.0)	5,988 (100.0)	5,988 (100.0)
Age, mean (SD), y	12.75 (1.41)	13.75 (1.41)	14.75 (1.41)
Gender			
Boys	3,013 (50.3)	3,013 (50.3)	3,013 (50.3)
Girls	2,975 (49.7)	2,975 (49.7)	2,975 (49.7)
Ethnicity			
Han	5,917 (98.9)	5,917 (98.9)	5,917 (98.9)
Other	71 (1.2)	71 (1.2)	71 (1.2)
Region			
Urban	2,864 (47.8)	2,869 (47.9)	2,875 (48.0)
Suburban	3,124 (52.2)	3,119 (52.1)	3,113 (52.0)
Living with father/mother			
Yes	5,831 (97.4)	5,823 (97.2)	5,863 (97.9)
No	157 (2.6)	165 (2.8)	125 (2.1)
Living on campus			
Yes	1,315 (22.0)	1,245 (20.8)	1,229 (20.5)
No	4,673 (78.0)	4,743 (79.2)	4,759 (79.5)
Education level			
Junior high school	5,095 (85.1)	4,949 (82.6)	4,611 (77.0)
Senior high school	893 (14.9)	1,039 (17.4)	1,377 (23.0)
BMI, mean (SD), kg/m^2^	21.55 (4.46)	21.72 (4.65)	22.10 (4.74)

Note: *SD* standard deviation; *BMI* body mass index; T1, year 2022; T2, year 2023; T3, year 2024

**Fig. 1:**
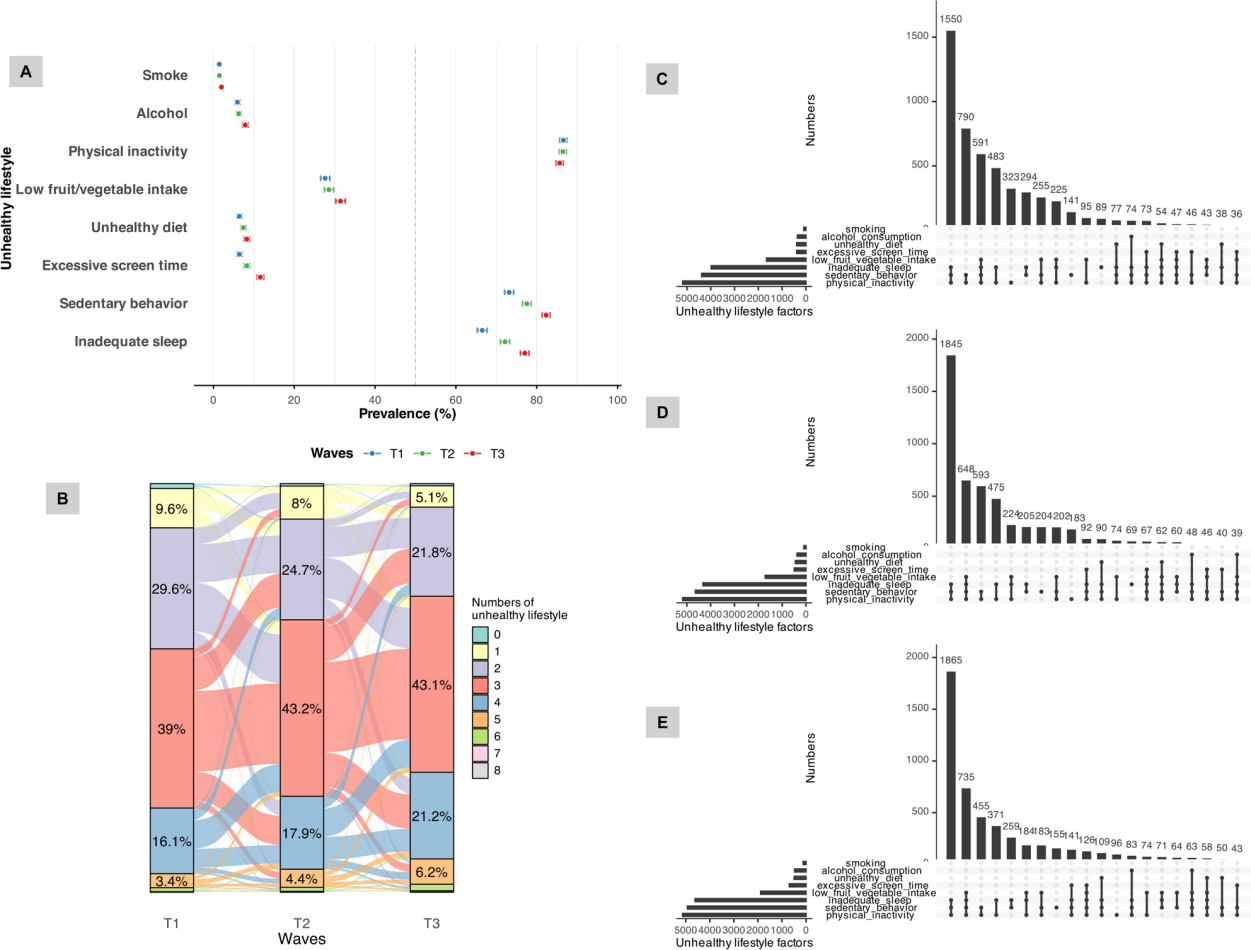
Prevalence, accumulation, and co-occurrence patterns of unhealthy lifestyle factors across three longitudinal waves. **A** Prevalence of eight unhealthy lifestyle factors at three time points: T1 (blue), T2 (green), and T3 (red). Data are presented as prevalence (%) with error bars indicating 95% confidence intervals. **B** Composition of the number of unhealthy lifestyle factors (ranging from 0 to 8) across waves T1, T2, and T3. The width of each flow represents the proportion of participants transitioning between different lifestyle burden levels, with colors indicating the number of unhealthy lifestyle factors. **C–E** Composition of lifestyle type combinations within each wave: T1 (C), T2 (D), and T3 (E). Each diagram illustrates the co-occurrence patterns and the corresponding number of participants with different unhealthy lifestyle factors. Due to space constraints, only the top 20 most frequent combinations of unhealthy lifestyle factors are presented. T1, year 2022; T2, year 2023; T3, year 2024

The descriptive statistics for unhealthy lifestyle factors across the three waves are shown (Fig. 1A; Additional file 1: Table S2). From T1 to T3, the prevalence of unhealthy lifestyle factors generally increased (except for physical inactivity), and these changes were statistically significant (all *p* < 0.05). Among the eight unhealthy lifestyle factors, smoking had the lowest prevalence (T1 and T2: 1.5%; T3: 2.0%), while physical inactivity was the most common (T1: 86.6%; T2: 86.5%; T3: 85.7%). More than half of the students exhibited physical inactivity, sedentary behavior, and inadequate sleep. The distribution of the number of unhealthy lifestyle factors across the three waves is shown in Fig. 1B. The percentage of participants reporting four or more unhealthy lifestyle factors increased over time. Detailed percentage information was provided in Additional file 1: Table S3. Figure 1C–E show the distribution of co-occurring unhealthy lifestyle factors at T1, T2, and T3. Across all three time points, the most prevalent combination of unhealthy lifestyle factors consisted of inadequate sleep, sedentary behavior, and physical inactivity. Notably, the proportion of adolescents exhibiting the co-occurrence of low fruit/vegetable intake, inadequate sleep, sedentary behavior, and physical inactivity increased significantly from T1 to T3 (*p* < 0.001).

Descriptive statistics for depressive symptoms and the number of unhealthy lifestyle factors at T1, T2, and T3 are shown in Table 2. At baseline, the mean depressive symptom score was 7.96 (SD = 7.53). The mean score increased to 8.09 (SD = 8.06) at T2 and further to 8.80 (SD = 8.31) at T3, showing a slight increase in depressive symptoms from T1 to T3 (*p* < 0.001). The mean number of unhealthy lifestyle factors was 2.74 (SD = 1.06) at T1, which increased to 2.88 (SD = 1.04) at T2 and further to 3.06 (SD = 1.07) at T3, suggesting a significant and gradual accumulation of unhealthy lifestyle factors across waves (all *p* < 0.001).

Table 2 shows the Spearman correlation results between the number of unhealthy lifestyle factors and depressive symptoms. Depressive symptoms showed statistically significant correlations across waves (ρ = 0.302–0.430, all *p* < 0.001). Similarly, the number of unhealthy lifestyle factors showed significant correlations over time (ρ = 0.224–0.320, all *p* < 0.001). At each wave, depressive symptoms were positively correlated with the number of unhealthy lifestyle factors (ρ = 0.093–0.282, all *p* < 0.001), suggesting that adolescents with a greater number of unhealthy lifestyle factors tended to report higher levels of depressive symptoms.

**Table 2:** Spearman correlations between depressive symptoms and the number of unhealthy lifestyle factors at T1, T2, and T3

	Mean ± SD	DS T1	DS T2	DS T3	UL T1	UL T2	UL T3
DS T1	7.96 ± 7.53	1					
DS T2	8.09 ± 8.06	0.430***	1				
DS T3	8.80 ± 8.31	0.302***	0.386***	1			
UL T1	2.74 ± 1.06	0.214***	0.143***	0.093***	1		
UL T2	2.88 ± 1.04	0.171***	0.267***	0.135***	0.320***	1	
UL T3	3.06 ± 1.07	0.126***	0.159***	0.282***	0.224***	0.271***	1

Note: *DS* depressive symptoms, *UL* unhealthy lifestyle, *SD* standard deviation; T1, year 2022; T2, year 2023; T3, year 2024

***, *p* < 0.001

### Longitudinal associations between the number of unhealthy lifestyle factors and depressive symptoms

Model fit indices and comparison results for the unconstrained (Model c) and constrained RI-CLPMs (Models d–f) are provided (Additional file 1: Table S4). After adjusting for both time-invariant and time-varying covariates, Model c showed that depressive symptoms at time T significantly predicted the number of unhealthy lifestyle factors at time T + 1, whereas the reverse cross-lagged paths were non-significant, with an acceptable model fit (RMSEA = 0.031; CFI = 0.948; TLI = 0.908; SRMR = 0.025). The constrained RI-CLPMs (Models d, e, and f) also showed the same unidirectional temporal pattern. Constraining only the autoregressive paths (Model d) significantly worsened the model fit. In contrast, constraining only the cross-lagged paths (Model e), or both the cross-lagged and autoregressive paths (Model f) did not lead to a significant deterioration in fit. Based on the model fit indices, the most constrained model (Model f) was selected as the final RI-CLPM (RMSEA = 0.030; CFI = 0.949; TLI = 0.916; SRMR = 0.026).

**Fig. 2:**
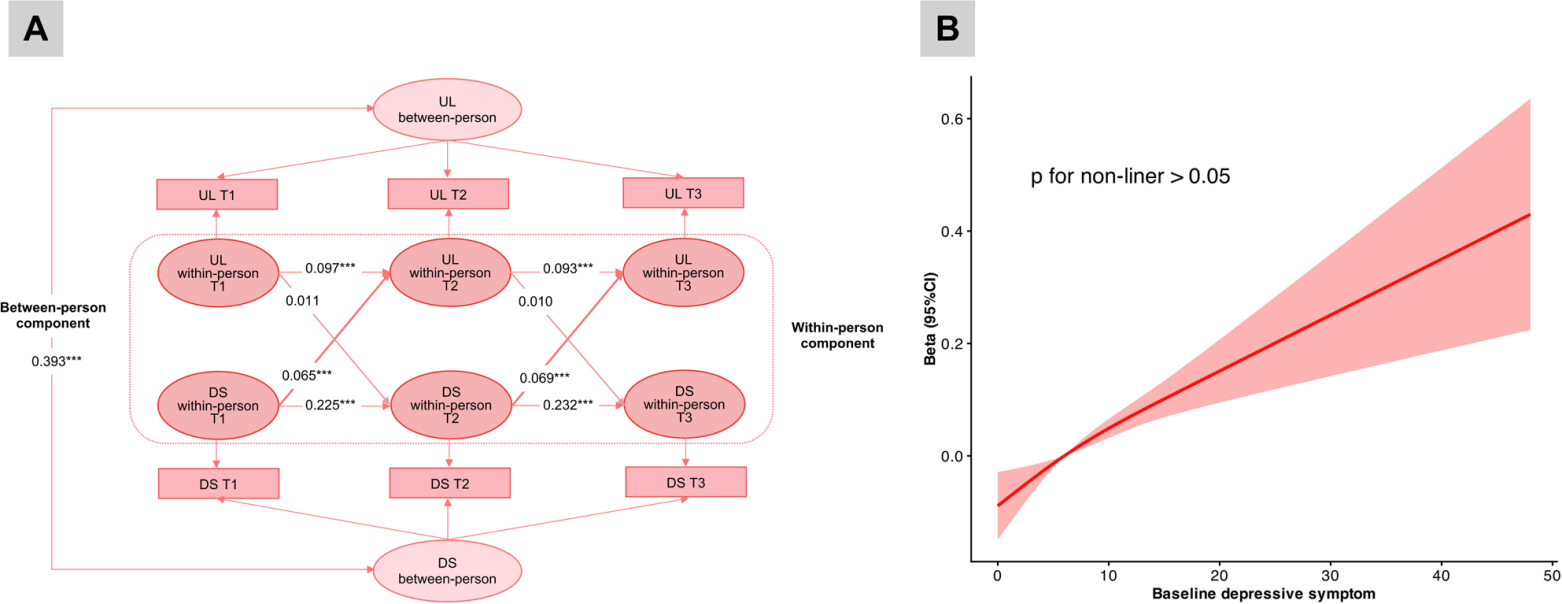
Longitudinal associations between depressive symptoms and the number of unhealthy lifestyle factors across three waves. **A** Random intercept cross-lagged panel model of depressive symptoms and the number of unhealthy lifestyle factors with autoregressive and cross-lagged paths constrained to be equal across time. The results indicate a unidirectional pathway: depressive symptoms at each time point significantly predict the number of unhealthy lifestyle factors at the subsequent time point (T→T + 1), whereas the reverse pathways were not significant. All covariates were estimated in the analysis but not shown. **B** Dose-response relationship between depressive symptoms at T1 and the number of unhealthy lifestyle factors at T3. The analysis shows a linear relationship between baseline depressive symptoms and subsequent accumulation of unhealthy lifestyle factors. The red line represents the adjusted β coefficient for the number of unhealthy lifestyle factors, and the shaded area indicates the 95% confidence interval. The model was adjusted for age z-score, gender, ethnicity, region, education level, BMI z-score, living with father/mother, living on campus, and the number of unhealthy lifestyle factors at T1. CES-D, the Center for Epidemiologic Studies Depression Scale; DS, depressive symptoms; UL, unhealthy lifestyle factors; T1, year 2022; T2, year 2023; T3, year 2024. ***p < 0.001

The final RI-CLPM results are shown in Fig. 2A. In the between-person component, the random intercepts of depressive symptoms and the number of unhealthy lifestyle factors were significantly and positively correlated, indicating a strong and stable association between individuals’ trait-level depressive symptoms and the number of unhealthy lifestyle factors (*r* = 0.393, *p* < 0.001). In the within-person component, depressive symptoms at time T had a significant, medium effect on the number of unhealthy lifestyle factors at time T + 1 (β = 0.065–0.069; all *p* < 0.001). Conversely, the number of unhealthy lifestyle factors at time T did not significantly predict depressive symptoms at time T + 1 (β = 0.010–0.011; all *p* > 0.05). These results were consistent across unconstrained and constrained models. Detailed unstandardized and standardized estimates for the autoregressive and cross-lagged paths of Model f are provided (Additional file 1: Table S5). The regression results of covariates are presented (Additional file 1: Table S6). The results of the sensitivity analyses further supported this unidirectional temporal pattern of associations between depressive symptoms and the number of unhealthy lifestyle factors, with acceptable model fit indices (Additional file 1: Table S7).

Furthermore, an RCS model was used to explore the non-linear relationship between depressive symptoms at T1 and the number of unhealthy lifestyle factors at T3. Based on the principle of minimum AIC, the RCS with three knots was selected. After adjusting for all covariates, a linear positive association was observed (Fig. 2B). The estimated regression coefficient for the number of unhealthy lifestyle factors increased steadily with higher levels of depressive symptoms.

### Depressive symptom trajectories

**Fig. 3:**
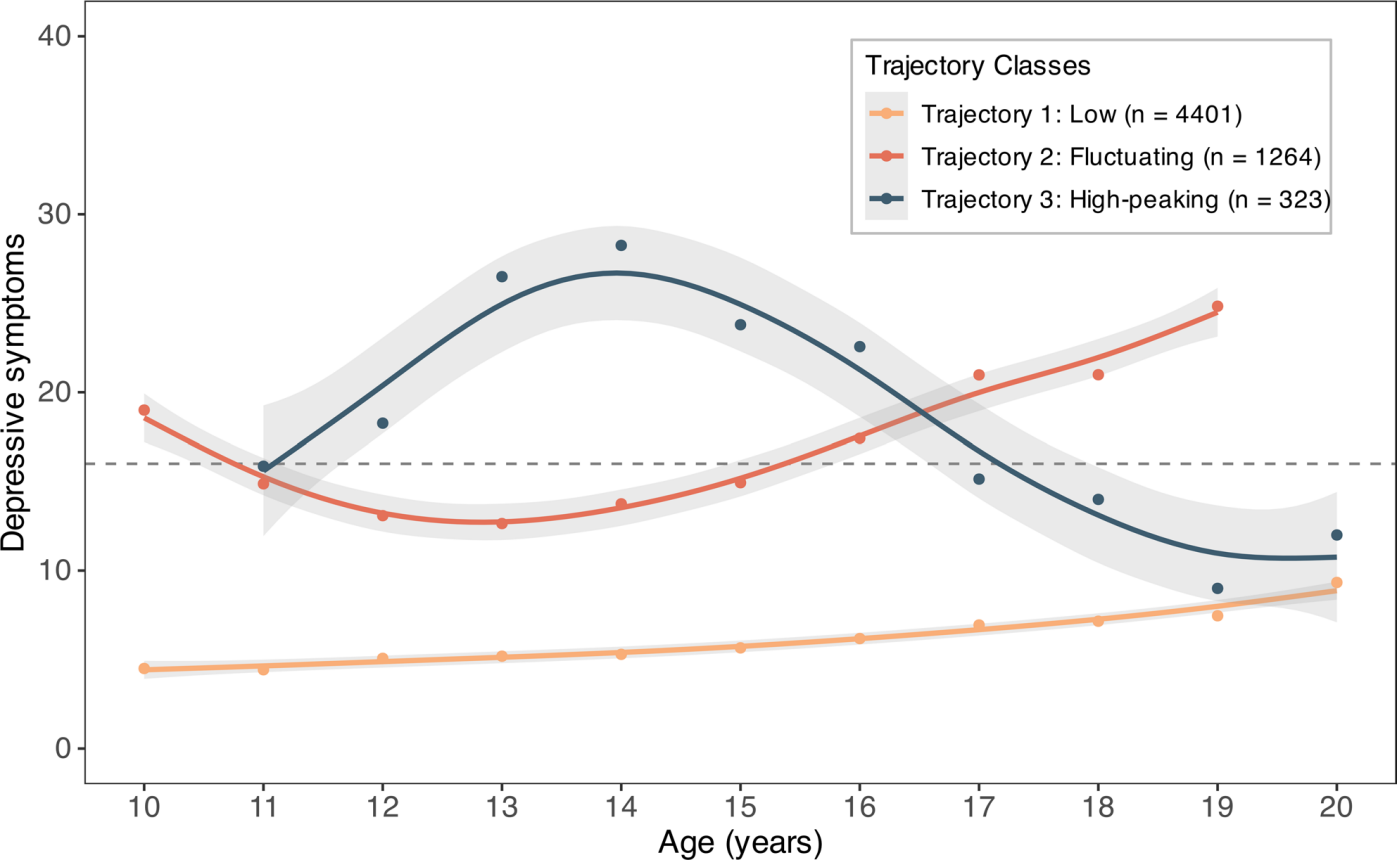
Trajectories of depressive symptoms over age. Three distinct depressive symptom trajectories were identified: “low” (*n* = 4,401), “fluctuating” (*n* = 1,264), and “high peaking” (*n* = 323)

The model fit indices for the LCMM are presented (Additional file 1: Table S8). Following the predefined criteria, a three-class trajectory model was selected as the final solution. Three depressive symptom trajectories over age were identified: “low” (*n* = 4,401, 73.5%), “fluctuating” (*n* = 1,264, 21.1%), and “high peaking” (*n* = 323, 5.4%) (Fig. 3). Detailed descriptions of the three depressive symptom trajectories are provided in Additional file 1: Table S9. The “low” trajectory reflected a relatively stable low-risk pattern of depressive symptoms across adolescence. The “fluctuating” trajectory suggested substantial instability in depressive symptoms, characterized by both early improvement and subsequent deterioration over time. The “high peaking” trajectory reflected an early-onset high-risk pattern, characterized by a pronounced peak in depressive symptoms during early adolescence followed by a gradual decline.

Demographic differences across depressive symptom trajectories are examined (Additional file 1: Table S10). Across the three time points, no significant differences were observed among depressive symptom trajectories in terms of age, ethnicity, education level, or BMI. In contrast, significant differences were found for gender, region, living with father/mother, and living on campus (except at baseline). Compared with the “low” trajectory group, adolescents in the “fluctuating” and “high peaking” trajectory groups had a higher proportion of girls, students attending urban schools, those not living with their parents, and those living on campus.

Trajectory group differences in the prevalence of specific unhealthy lifestyle factors and the overall number of unhealthy lifestyle factors across the three time points (T1, T2, and T3) were statistically significant for all variables except sedentary behavior (Additional file 1: Table S10). Participants in the “high peaking” trajectory group had higher proportions of smoking, alcohol consumption, unhealthy diet, and excessive screen time, and this group also reported the highest mean number of unhealthy lifestyle factors. Participants in the “fluctuating” trajectory group had higher rates of physical inactivity, low fruit/vegetable intake, and inadequate sleep.

### The relationship between depressive symptom trajectories and the number of unhealthy lifestyle factors

For analyses with the number of unhealthy lifestyle factors as the outcome, the data showed no evidence of overdispersion (dispersion = 0.326). Therefore, the primary analysis employed a GLMM with a Poisson distribution, adjusting for covariates (Table 3). The results indicated that, compared with the “low” trajectory group, participants in both the “fluctuating” and “high peaking” trajectory groups had significantly higher expected counts of unhealthy lifestyle factors at T3 (fluctuating: AIRR = 1.128, 95% CI: 1.088 to 1.171, FDR-adjusted *p* < 0.001; high peaking: AIRR = 1.162, 95% CI: 1.092 to 1.236, FDR-adjusted *p* < 0.001). However, the difference in the number of unhealthy lifestyle factors between the “fluctuating” and “high peaking” trajectory groups was not statistically significant (AIRR = 1.030, 95% CI: 0.965 to 1.100, FDR-adjusted *p* = 0.374).

**Table 3:** Primary model of the association between depressive symptom trajectories and the number of unhealthy lifestyle factors at T3, controlling for covariates at T1

Variables	GLMM (Poisson)	
	AIRR (95%CI)	*P* value
Intercept	2.412 (2.039, 2.854)	< 0.001
Age, z-score	1.038 (1.010, 1.067)	0.007
Boys	1.014 (0.985, 1.045)	0.353
Han	1.003 (0.876, 1.147)	0.971
Urban	0.980 (0.940, 1.022)	0.350
Senior high school	0.985 (0.908, 1.068)	0.709
BMI, z-score	0.996 (0.981, 1.010)	0.553
Living with father/mother	1.048 (0.956, 1.149)	0.314
Living on campus	1.043 (0.998, 1.089)	0.060
Number of unhealthy lifestyle factors at T1	1.056 (1.041, 1.071)	< 0.001
Depressive symptoms trajectory classes		
Traj. 2 : Traj. 1	1.128 (1.088, 1.171)	< 0.001
Traj. 3 : Traj. 1	1.162 (1.092, 1.236)	< 0.001
Traj. 3 : Traj. 2	1.030 (0.965, 1.100)	0.374

Note: *AIRR* adjusted incidence rate ratio; Traj. 1, Low; Traj. 2, Fluctuating; Traj. 3, High peaking; *GLMM* (Poisson) Poisson generalized linear mixed model; *BMI* body mass index; T1, year 2022; T3, year 2024

Sensitivity analyses yielded similar findings (Additional file 1: Table S11). Model S1 demonstrated that higher baseline depressive symptom scores were significantly associated with a greater number of unhealthy lifestyle factors at T3. Model S2 (using a linear mixed model) and Model S3 (using a binomial GLMM for the dichotomized lifestyle variable) also confirmed that participants in the “fluctuating” and “high peaking” trajectory groups had more unhealthy lifestyle factors compared to the “low” trajectory group, with no significant difference between the two higher-risk groups.

When the outcome variable was depressive symptoms at T3, significant overdispersion was present (dispersion = 2.243, *p* < 0.001). The Vuong test indicated that a zero-inflated negative binomial model provided a better fit. Consequently, a zero-inflated negative binomial model was used to explore the association between the number of unhealthy lifestyle factors at T1 and depressive symptoms at T3. Analyses investigating the associations between the number of unhealthy lifestyle factors at T1 and both depressive symptoms at T3 and depressive symptom trajectories yielded non-significant results (Additional file 1: Table S12). These findings were consistent with the results from the RI-CLPMs, suggesting that the baseline number of unhealthy lifestyle factors was not significantly associated with later depressive symptoms or depressive symptom trajectories in this study.

## Discussion

To our knowledge, this is the first study to investigate the longitudinal relationship between the cumulative number of unhealthy lifestyle factors, depressive symptoms, and depressive symptom trajectories among Chinese adolescents. After accounting for both between- and within-person differences, our findings showed that higher levels of depressive symptoms were associated with subsequent increases in the number of unhealthy lifestyle factors over time, whereas the number of unhealthy lifestyle factors were not significantly associated with subsequent depressive symptoms. This suggests that, in the interplay between psychological and behavioral factors during adolescence, depressive symptoms may temporally precede behavioral risk accumulation. In addition, adolescents following “fluctuating” or “high peaking” trajectories of depressive symptoms accumulated more unhealthy behaviors, further highlighting the long-term impact of depressive symptom trajectories on lifestyle behaviors. Overall, this study provides evidence of a unidirectional temporal pattern between adolescent mental health and unhealthy lifestyle risks, offering potential implications for developing interventions that directly target depressive symptoms as a means of reducing the later accumulation of unhealthy lifestyle behaviors.

After isolating stable between-person differences, this study found that worsening depressive symptoms in adolescents showed a unidirectional association with subsequent increases in unhealthy lifestyle factors. Sensitivity analyses further supported this unidirectional temporal pattern of associations, indicating that the observed association was robust and not driven by correlations between specific unhealthy lifestyle behaviors (e.g., inadequate sleep and physical inactivity) and relevant items of the depressive symptom scale. This pattern differs from the relationships observed in some adult studies, where unhealthy lifestyles have been shown to influence depression. Prior evidence indicates that adults with unhealthy lifestyles are at higher risk of developing depression. For example, one study reported significant associations between multiple lifestyle risk factors and depression [6], while a prospective cohort study found that all lifestyle risk behaviors were linked to increased depression risk in adults, including the clustering of 2–4 unhealthy lifestyle behaviors [51]. A bidirectional Mendelian randomization study utilizing the UK Biobank (UKB) database revealed a bidirectional association between physical activity, sedentary time, and depression [52]. Another Mendelian randomization study found that higher levels of physical activity reduced the risk of depression in the general population, whereas the causal effect of depression on physical activity was relatively weak [53]. However, one study that conducted both a three-wave longitudinal analysis among university students and a unidirectional Mendelian randomization analysis did not identify causal associations between consumption of sweets, fruits, or vegetables, or physical activity and depression [54]. In addition, a literature review of two-sample Mendelian randomization studies found no evidence supporting a causal relationship between diet and depression [55]. Among adolescents, one study reported that adherence to a greater number of healthy lifestyle behaviors was associated with lower depressive symptom scores one year later [56]. Another longitudinal study in adolescents found that unhealthy behaviors during adolescence were associated with depression risk in early adulthood, but the association became non-significant after adjusting for relevant covariates [57].

Taken together with our finding that depressive symptoms showed a unidirectional temporal association with later increases in the number of unhealthy lifestyle behaviors, these discrepancies may be explained by methodological and population differences. First, much of the existing evidence was derived from adult populations, in whom lifestyle behaviors tended to be more stable. In contrast, adolescents are in a critical developmental period characterized by heightened emotional reactivity and still-maturing self-regulatory systems, making their lifestyle behaviors more susceptible to short-term fluctuations in emotional states. Second, different analytical approaches capture distinct aspects of the depression–lifestyle relationship. Conventional regression and prospective cohort analyses primarily estimate between-individual associations at the population level. Mendelian randomization studies, by contrast, are designed to infer lifelong causal effects of genetically proxied exposures and may be less sensitive to developmentally specific or time-varying behavioral changes that are particularly salient during adolescence. By applying the RI-CLPM, the present study isolates within-individual temporal processes after accounting for stable between-person differences, thereby suggesting that short-term worsening of depressive symptoms may precede the accumulation of unhealthy lifestyle behaviors during adolescence. Collectively, these differences in study populations and causal inference frameworks may contribute to the heterogeneous and inconsistent findings observed in the literature. Supporting this view, a study reported that prior-year depressive symptom levels and their rate of change were significantly associated with increased unhealthy dietary behaviors [58]. Consistent with our findings, this study suggests that in adolescence, the clustering of multiple unhealthy behaviors may be more closely linked to deteriorating mood than indicated by the reverse pathway in the present analyses. From the perspective of behavioral mechanisms, depressed adolescents may choose high-calorie foods as a coping strategy [59]. On the other hand, depressive symptoms may reduce adolescents’ self-efficacy [60, 61]. Previous studies have also found that adolescents with higher levels of self-efficacy tend to consume more fruits and vegetables and engage in more physical activity [60–62]. Conversely, adolescents with more severe depressive symptoms find it harder to maintain healthy lifestyle behaviors, including disordered eating behaviors and reduced physical activity, thereby increasing the number of unhealthy lifestyle factors [63, 64].

In this study, depressive symptoms were assessed using the CES-D, which captures depressive symptoms experienced during the past week, and most unhealthy lifestyle behaviors were measured using a similar short-term reference period. Accordingly, the constructs examined in this study primarily reflect short-term depressive symptoms and concurrent behavioral patterns rather than stable traits or long-term lifestyle habits. Within this framework, the longitudinal associations observed suggest that increases in depressive symptoms at one wave tend to precede subsequent changes in adolescents’ behavioral states at the next wave. At the same time, the use of short-term measures may limit sensitivity to more gradual, cumulative effects of lifestyle behaviors on depressive symptoms, which may unfold over longer periods and require sustained exposure to be detected. These findings have important public health implications: to prevent adolescents from adopting unhealthy lifestyles, the key lies not merely in modifying specific behaviors, but in early identification and intervention targeting the worsening trend of depressive symptoms. Psychosocial or behavioral interventions that directly target depressive symptoms, including negative affect, low motivation, and emotional distress, may represent an important complement to traditional lifestyle-focused health promotion strategies, as they can not only address unhealthy lifestyle behaviors directly but also help prevent the emergence and accumulation of such behaviors by reducing depressive symptoms.

Using RCS modeling, this study identified an approximately linear relationship between depressive symptoms and the number of unhealthy lifestyle factors, indicating that each unit increase in depressive symptoms is associated with a proportional rise in lifestyle-related risks. This pattern differs from findings reported in some adult studies. For example, one study observed a nonlinear relationship in adults, where depression risk increased sharply only after the accumulation of unhealthy behaviors reached a certain threshold [27]. Another study among adults hospitalized for depression also reported a linear association between depression recurrence and behaviors such as diet and sleep, whereas smoking and alcohol use exhibited nonlinear effects, with risk rising significantly only at higher exposure levels [65]. These differences between adolescents and adults may reflect fundamental developmental distinctions. Adolescents’ still-maturing emotion regulation and self-control systems render their self-management capacities more vulnerable to being undermined by depressive symptoms. Consequently, worsening depressive symptoms may be reflected simultaneously across multiple behavioral domains, such as physical activity, sleep, diet, and screen use, without requiring a specific threshold to be triggered. Therefore, in both school and public health practice, intervention efforts should be shifted earlier, employing integrated strategies, such as emotion regulation training and sleep support, to proactively disrupt this linear trajectory of accumulating behavioral risks.

Through the interaction of social, psychological, and biological factors, children and adolescents may follow distinct developmental trajectories across different periods of development [66]. The three depressive symptom trajectories identified in this study are broadly consistent with findings from previous longitudinal research in adolescents, wherein the majority follow a “consistently low” pattern, while a minority exhibit “high peaking” or “fluctuating” symptom trajectories [67, 68]. Adolescents in the “high peaking” group may experience brief yet pronounced worsening of depressive symptoms, which may be linked to acute stress exposure or critical life events. In contrast, those in the “fluctuating” trajectory show a period of symptom remission followed by a subsequent rise, suggesting greater susceptibility to persistent or recurrent stressors. Previous studies have indicated that adolescents in high depressive symptom trajectories are closely linked to difficulties in emotion regulation, increased risk behaviors, and insufficient physical activity [68, 69]. This further highlights that depression does not follow a linear developmental course; some adolescents exhibit higher vulnerability to recurrence and symptom rebound, necessitating long-term monitoring and sustained support for vulnerable adolescents.

Both the “high peaking” and “fluctuating” trajectory groups in our study showed a significant accumulation of unhealthy lifestyle risks. This finding contrasts with a study using group-based trajectory modeling (GBTM), which reported no consistent associations between depressive symptom trajectories and individual unhealthy behaviors—except in the persistently high depressive symptom group [31]. This discrepancy underscores that focusing on single behaviors may underestimate the comprehensive health impact of depression, whereas the cumulative number of unhealthy behaviors may more sensitively capture the true effect of depressive symptoms on unhealthy lifestyles. Furthermore, our study observed that although adolescents in the “fluctuating” group showed a decline in depressive symptoms during mid-adolescence, their symptom levels subsequently rose again. This has important public health implications: the timing of the first depressive episode may be less critical than the need for prompt assessment and intervention as soon as depressive signs emerge [70]. The “drop-and-rise” pattern observed in the “fluctuating” group suggests that adolescent health promotion programs should not be discontinued based on temporary symptom improvement; otherwise, when new stressors occur, depressive symptoms and associated unhealthy behaviors may rapidly rebound. Thus, adolescents following “fluctuating” or “high peaking” depressive trajectories should be recognized as a long-term at-risk population and prioritized for sustained mental health services. Consistent, long-term, multifaceted monitoring and interventions targeting depressive symptoms are essential to prevent recurrent depressive symptoms and their behavioral consequences. For example, schools should not merely complete annual health behavior surveys but also establish adequately resourced psychological service centers, staffed with a sufficient number of certified counselors, to provide persistently accessible counseling and early identification channels. Both teachers and parents should remain attentive to adolescents’ negative emotions, foster open communication, and offer timely reassurance and support at the early signs of depressed moods. Society as a whole should strive to reduce the stigma associated with mental health issues, enhance public awareness through media, and ensure the long-term availability and accessibility of community-based mental health resources.

Regarding covariates, the results showed that, after adjustment for other variables, only baseline age was significantly associated with the number of unhealthy lifestyle behaviors during the follow-up period. Consistent with previous findings, unhealthy lifestyle behaviors tend to accumulate with increasing age [71], and previous studies have also reported a higher prevalence of unhealthy lifestyle behaviors among older adolescents [71]. With respect to depressive symptom trajectories, this study found that girls were more likely to belong to the “fluctuating” and “high peaking” depressive trajectories, particularly the “high peaking” trajectory. This pattern is consistent with prior evidence indicating higher levels of depressive symptoms among girls [30]. During adolescence, girls tend to place greater emphasis on close and harmonious interpersonal relationships and may experience higher levels of peer-related stress, which may increase their vulnerability to depressive symptoms [72]. In addition, students attending urban schools are more likely to be classified as belonging to high-risk depressive symptom trajectories. Adolescents in urban school settings may be exposed to greater academic pressure and competition, a finding that aligns with previous research [73]. One longitudinal study reported that, among urban adolescents, a subgroup experienced more severe depressive symptoms with a rapid increase during adolescence, whereas such a pattern was not observed among their rural counterparts [74]. However, other studies have reported no significant differences in depressive symptoms between urban and rural students [75]. Furthermore, adolescents who had not been living with either their father or mother in the past six months were more likely to belong to high-risk depressive trajectories. Previous research has shown that a lack of maternal companionship is associated with higher levels of depressive symptoms among adolescents [76], and adolescents whose mothers were absent for more than six months have been found to have an increased risk of depression [77, 78]. Students living on campus were also more likely to be classified as belonging to high-risk depressive trajectories, which is consistent with multiple studies reporting a higher prevalence of depressive symptoms among adolescents living in boarding schools [79, 80].

This study has several limitations. First, the data were collected annually through school-based surveys, resulting in relatively long measurement intervals that may not capture rapid, short-term fluctuations in adolescents’ depressive symptoms and lifestyle behaviors. Annual assessments allow the examination of year-to-year associations but may not fully reflect the uniquely volatile developmental context of adolescence, a period characterized by rapid neurobiological maturation, emerging self-regulation, and major school transitions. During this stage, depressive symptoms and lifestyle behaviors may fluctuate substantially in response to proximal stressors such as examination pressure, changes in school environments, peer relationship dynamics, and puberty-related developmental changes. As a result, annual measurement intervals may miss short-term, stress-triggered fluctuations that are typical in adolescence (e.g., during exam periods or school term transitions), potentially confounding or moderating the observed within-person associations between depressive symptoms and the accumulation of unhealthy behaviors and biasing inferences about their temporal directionality. Future research could benefit from more frequent assessments, such as semester-based or monthly follow-ups, or the use of ecological momentary assessment (EMA) approaches, to better capture short-term dynamics and temporal sequencing. In addition, future research should explicitly model developmental transitions and contextual stressors to better align measurement strategies with the dynamic nature of adolescent development. Second, lifestyle behaviors were predominantly self-reported, which may be subject to recall bias or social desirability bias. Future research may incorporate objective or device-based measures, such as accelerometers for physical activity or digital logs for screen use, and triangulate self-reports with parent or teacher reports to improve measurement validity. Third, the composite lifestyle indicator was based on a simple count of behaviors rather than a weighted model, which does not account for differential health risks associated with different behaviors. Subsequent studies could apply weighted indices, validated behavior-specific scales, or latent variable approaches to better reflect the relative contributions of specific behaviors to mental health outcomes. Fourth, due to the constraints of the questionnaire design, smoking and alcohol use were only recorded as binary variables (ever/never use), limiting the ability to capture frequency or persistence and potentially underestimating subtle changes in these behaviors among a subset of high-risk adolescents. Future surveys should assess the frequency and duration of tobacco, alcohol, and other substance use to better characterize patterns and trajectories of use. Fifth, although several time-varying and time-invariant covariates were adjusted for, unmeasured confounding may still exist. Sixth, although the temporal ordering observed in the present analyses supports the interpretation that depressive symptoms may precede later accumulation of unhealthy lifestyle factors, alternative explanations, such as residual confounding, shared underlying vulnerabilities, and the possibility that lifestyle effects on depressive symptoms unfold over longer periods than captured here, cannot be excluded. Future studies should further investigate the temporal directionality between depressive symptoms and the accumulation of unhealthy lifestyle factors in adolescents by using more frequent assessments and incorporating additional psychosocial, family, and school-level factors to better address alternative explanations and residual confounding. Finally, although the present sample was drawn from a large school-based adolescent population, its generalizability to adolescents from other cultural and socioeconomic contexts may be limited. As indicated by the attrition analysis, the longitudinal sample is likely biased toward younger students due to the school-based sampling design, whereby older students have a lower probability of being retained across waves. Furthermore, patterns of depressive symptoms and lifestyle behaviors are shaped by educational systems, social norms, and environmental exposures, which may differ across regions and countries. Therefore, caution is warranted when generalizing the observed within-individual dynamics, particularly to older adolescents or to populations in distinct cultural and socioeconomic contexts.

## Conclusions

This study suggests that depressive symptoms may temporally precede subsequent increases in the number of unhealthy lifestyle behaviors among adolescents, whereas the reverse direction was not supported in the present analyses. Adolescents following distinct depressive symptom trajectories exhibit significant differences in behavioral risks, with those in the “fluctuating” and “high peaking” groups showing a greater tendency toward sustained accumulation of unhealthy behaviors throughout adolescence. These findings suggest that, in both clinical and public health practice, psychosocial or behavioral interventions targeting underlying depressive symptoms may help reduce the risk of adopting unhealthy lifestyles during adolescence. In addition, long-term depressive symptom monitoring and early intervention are essential to prevent recurrent depressive symptoms and their associated behavioral consequences.

## Supplementary Information

Below is the link to the electronic supplementary material.

Supplementary Material 1: Additional File 1. All supplementary tables and figures, including Figure S1 and Tables S1–S12.

## Data Availability

The datasets analyzed in this study were obtained from the Surveillance of Students’ Common Diseases and Health Influencing Factors and contain sensitive information on minors. Public deposition of the raw data is not permitted due to ethical and legal restrictions, including requirements for adolescent privacy protection, confidentiality agreements, and institutional/provincial regulations governing access to surveillance data. De-identified individual-level data may be made available for non-commercial, scientific research purposes under controlled access, subject to (1) a reasonable request describing the research aims and analysis plan, (2) approval by the Shandong Center for Disease Control and Prevention (Shandong CDC) and relevant ethics/data governance procedures, and (3) execution of a data use agreement that prohibits any attempt at re-identification and any onward sharing/redistribution of the data. Requests should be directed to the corresponding data custodian at Shandong CDC: Dr. Lianlong Yu (Email: lianlong00a@163.com). The expected timeframe for an initial response to access requests is within 2–4 weeks. Approved access will be provided via on-site review and analysis at the Shandong CDC in accordance with institutional and provincial ethical guidelines; the data cannot be removed from the secure environment. The analysis code of this study is publicly available at: https://github.com/Afei-Qin/Longitudinal-depressive-symptoms-and-the-number-of-unhealthy-lifestyle-factors-in-adolescents.

## Abbreviations

RI-CLPM: The random intercepts cross-lagged panel model
GLMMs: Generalized linear mixed models
WHO: World Health Organization
AUD: Alcohol use disorder
MVPA: Moderate-to-vigorous physical activity
NHANES: National Health and Nutrition Examination Survey
SSCDHIF: Surveillance of Students’ Common Diseases and Health Influencing Factors
CES-D: The 20-item Center for Epidemiologic Studies Depression Scale
MLR: Maximum likelihood estimator with robust standard errors
CFI: Comparative Fit Index
TLI: Tucker-Lewis Index
RMSEA: Root Mean Square Error of Approximation
SRMR: Standardized Root Mean Square Residual
BMI: Body mass index
CI: Confidence interval
SD: Standard deviation
AIC: Akaike Information Criterion
BIC: Bayesian Information Criterion
S–Bχ²: Satorra-Bentler scaled chi-square difference test
FIML: Full information maximum likelihood
RCS: Restricted cubic spline
LCMM: Latent class linear mixed model
APPA: Average Posterior Probability of Assignment
OCC: Odds of Correct Classification
AIRRs: Adjusted incidence rate ratios
LMMs: Linear mixed models
AORs: Adjusted odds ratios
FDR: False discovery rate
EMA: Ecological momentary assessment
