# Supplementary material for: Is there a bidirectional relationship between the number of unhealthy lifestyle factors and depressive symptoms in adolescents? Evidence from a longitudinal study
Source: BMC Med. 2026 Jun 10;24:447. doi: 10.1186/s12916-026-04984-9 (PMC13480264; doi:10.1186/s12916-026-04984-9)
Supplement: Supplementary file 1 [file 12916_2026_4984_MOESM1_ESM.docx]

**Supplementary material**

[Figure S1 Participant flow diagram across the three survey waves	1](#_Toc231234711)

[Table S1 Comparison of demographic variables between attrition and follow-up groups across time points	2](#_Toc231234712)

[Table S2 Prevalence of unhealthy lifestyle factors across three waves and pairwise comparisons among adolescents (N = 5,988)	4](#_Toc231234713)

[Table S3 Distributions of numbers of unhealthy lifestyle factors across three waves	6](#_Toc231234714)

[Table S4 Model fit indices and model comparisons for random intercept cross-lagged panel models	7](#_Toc231234715)

[Table S5 Non-standardized and standardized estimates for the cross-lagged and autoregressive paths of the final random intercept cross-lagged panel model (Model f)	8](#_Toc231234716)

[Table S6 Associations of covariates with depressive symptoms and the number of unhealthy lifestyle factors in the final random intercept cross-lagged panel model (Model f)	9](#_Toc231234717)

[Table S7 Standardized cross-lagged path estimates and model fit indices from sensitivity analyses using random-intercept cross-lagged panel models	11](#_Toc231234718)

[Table S8 Latent class linear mixed model selection	13](#_Toc231234719)

[Table S9 Detailed descriptions of three depressive symptom trajectories	14](#_Toc231234720)

[Table S10 Differences in demographic variables and unhealthy lifestyle factors at the three waves across depressive symptom trajectories	15](#_Toc231234721)

[Table S11 Sensitivity analyses of the association between depressive symptom trajectories, depressive symptoms at T1, and the number of unhealthy lifestyle factors at T3	20](#_Toc231234722)

[Table S12 Associations of the number of unhealthy lifestyle factors at T1 with depressive symptoms at T3 and depressive symptom trajectories	21](#_Toc231234723)

# Figure S1 Participant flow diagram across the three survey waves

6,165 longitudinal

participants from T1 to T3

25,625

follow-up at T3

128,852

attrition at T2

156,281

participants at T3

130,656

new participants at T3

147,011

participants at T1

2 repeated cases at T1

23,210

follow-up at T2

123,799

attrition at T1

131,267

new participants at T2

**Year 2022:** 147,011 adolescents

**Year 2023:** 154,477 adolescents

**Year 2024:** 156,281 adolescents

Note: T1, year 2022; T2, year 2023; T3, year 2024.

# Table S1 Comparison of demographic variables between attrition and follow-up groups across time points

| Variables | T1: 2022 → T2: 2023 | | | | T2: 2023 → T3: 2024 | | | |
| --- | --- | --- | --- | --- | --- | --- | --- | --- |
|  | Attrition [mean±SD/n (%)] | Follow-up [mean±SD/n (%)] | *χ*^2^/*F* | *P* value | Attrition [mean±SD/n (%)] | Follow-up [mean±SD/n (%)] | *χ*^2^/*F* | *P* value |
| Number | 123,799 (100.00) | 23,210 (100.00) |  |  | 128,852 (100.00) | 25,625 (100.00) |  |  |
| Age | 14.86±1.87 | 13.74±1.71 | 7225.000 | < 0.001 | 14.59±1.85 | 13.46±1.65 | 7225.0 | < 0.001 |
| Gender |  |  | 0.969 | 0.325 |  |  | 7.481 | 0.006 |
| Boys | 63,989 (51.69) | 12,079 (52.04) |  |  | 67,888 (52.69) | 13,261 (51.75) |  |  |
| Girls | 59,810 (48.31) | 11,131 (47.96) |  |  | 60,964 (47.31) | 12,364 (48.25) |  |  |
| Ethnicity |  |  | 10.640 | 0.001 |  |  | 10.926 | < 0.001 |
| Han | 122,064 (98.60) | 22,948 (98.87) |  |  | 127,275 (98.78) | 25,246 (98.52) |  |  |
| Other | 1,735 (1.40) | 262 (1.13) | 14.309 | < 0.001 | 1,577 (1.22) | 379 (1.48) |  |  |
| Region |  |  |  |  |  |  | 127.844 | < 0.001 |
| Urban | 61,651 (49.80) | 11,873 (51.15) |  |  | 67,089 (52.07) | 12,351 (48.2) |  |  |
| Suburban | 62,148 (50.20) | 11,337 (48.85) |  |  | 61,763 (47.93) | 13,274 (51.8) |  |  |
| Living with father/mother |  |  | 4.277 | 0.039 |  |  | 1.384 | 0.239 |
| Yes | 119,818 (96.91) | 22,519 (97.17) |  |  | 124,999 (97.04) | 24,899 (97.17) |  |  |
| No | 3,823 (3.09) | 657 (2.83) |  |  | 3,818 (2.96) | 724 (2.83) |  |  |
| Missing values (n) | 158 | 34 |  |  | 35 | 2 |  |  |
| Living on campus |  |  | 715.111 | < 0.001 |  |  | 623.826 | < 0.001 |
| Yes | 51,668 (41.74) | 7,509 (32.35) |  |  | 50,912 (39.51) | 7,998 (31.21) |  |  |
| No | 72,131 (58.26) | 15,701 (67.65) |  |  | 77,940 (60.49) | 17,627 (68.79) |  |  |
| Education level |  |  | 2699.072 | < 0.001 |  |  | 3254.242 | < 0.001 |
| Junior high school | 65,892 (53.22) | 16,634 (71.67) |  |  | 67,220 (52.17) | 18,339 (71.57) |  |  |
| Senior high school | 57,907 (46.78) | 6,576 (28.33) |  |  | 61,632 (47.83) | 7,286 (27.43) |  |  |
| BMI | 22.46±4.65 | 22.11±4.65 | 112.700 | < 0.001 | 22.28±4.79 | 21.74±4.70 | 112.7 | < 0.001 |
| Missing values (n) | 4 | 0 |  |  | 32 | 1 |  |  |

Note: n, number; SD, standard deviation; BMI, body mass index; T1, year 2022; T2, year 2023; T3, year 2024.

# Table S2 Prevalence of unhealthy lifestyle factors across three waves and pairwise comparisons among adolescents (N = 5,988)

| Unhealthy lifestyle factors | Waves | Yes | | | No [n (%)] | McNemar’s test |
| --- | --- | --- | --- | --- | --- | --- |
|  |  | N (%) | LCI | UCI |  |  |
| Smoking | T1 | 87 (1.5) | 1.17 | 1.80 | 5,901 (98.5) | T2 vs. T1: p = 0.858  T3 vs. T1: p = 0.012  T3 vs. T2: p = 0.017 |
|  | T2 | 89 (1.5) | 1.20 | 1.83 | 5,899 (98.5) |  |
|  | T3 | 119 (2.0) | 1.66 | 2.38 | 5,869 (98.0) |  |
| Alcohol consumption | T1 | 356 (5.9) | 5.37 | 6.58 | 5,632 (94.1) | T2 vs. T1: p = 0.394  T3 vs. T1: p < 0.001  T3 vs. T2: p < 0.001 |
|  | T2 | 374 (6.2) | 5.65 | 6.90 | 5,614 (93.8) |  |
|  | T3 | 472 (7.9) | 7.22 | 8.60 | 5,516 (92.1) |  |
| Physical inactivity | T1 | 5,185 (86.6) | 85.7 | 87.4 | 803 (13.4) | T2 vs. T1: p = 0.825  T3 vs. T1: p = 0.114  T3 vs. T2: p = 0.157 |
|  | T2 | 5,178 (86.5) | 85.6 | 87.3 | 810 (13.5) |  |
|  | T3 | 5,131 (85.7) | 84.8 | 86.6 | 857 (14.3) |  |
| Low fruit/vegetable intake | T1 | 1,656 (27.7) | 26.5 | 28.8 | 4,332 (72.3) | T2 vs. T1: p = 0.177  T3 vs. T1: p < 0.001  T3 vs. T2: p < 0.001 |
|  | T2 | 1,712 (28.6) | 27.5 | 29.8 | 4,276 (71.4) |  |
|  | T3 | 1,883 (31.4) | 30.3 | 32.6 | 4,105 (68.6) |  |
| Unhealthy diet | T1 | 384 (6.4) | 5.81 | 7.07 | 5,604 (93.6) | T2 vs. T1: p = 0.021  T3 vs. T1: p < 0.001  T3 vs. T2: p = 0.059 |
|  | T2 | 443 (7.4) | 6.75 | 8.10 | 5,545 (92.6) |  |
|  | T3 | 493 (8.2) | 7.56 | 8.97 | 5,495 (91.8) |  |
| Excessive screen time | T1 | 385 (6.4) | 5.83 | 7.09 | 5,603 (93.6) | T2 vs. T1: p < 0.001  T3 vs. T1: p < 0.001  T3 vs. T2: p < 0.001 |
|  | T2 | 493 (8.2) | 7.56 | 8.97 | 5,495 (91.8) |  |
|  | T3 | 695 (11.6) | 10.8 | 12.5 | 5,293 (88.4) |  |
| Sedentary behavior | T1 | 4,382 (73.2) | 72.0 | 74.3 | 1,606 (26.8) | T2 vs. T1: p < 0.001  T3 vs. T1: p < 0.001  T3 vs. T2: p < 0.001 |
|  | T2 | 4,645 (77.6) | 76.5 | 78.6 | 1,343 (22.4) |  |
|  | T3 | 4,930 (82.3) | 81.3 | 83.3 | 1,058 (17.7) |  |
| Inadequate sleep | T1 | 3,983 (66.5) | 65.3 | 67.7 | 2,005 (33.5) | T2 vs. T1: p < 0.001  T3 vs. T1: p < 0.001  T3 vs. T2: p < 0.001 |
|  | T2 | 4,321 (72.2) | 71.0 | 73.3 | 1,667 (27.8) |  |
|  | T3 | 4,614 (77.1) | 76.0 | 78.1 | 1,374 (22.9) |  |

Note: LCI, lower confidence interval; UCI, upper confidence interval; T1, year 2022; T2, year 2023; T3, year 2024.

# Table S3 Distributions of numbers of unhealthy lifestyle factors across three waves

| Numbers of unhealthy lifestyle factors | T1 | T2 | T3 |
| --- | --- | --- | --- |
| 0 | 70 (1.2%) | 35 (0.6%) | 32 (0.5%) |
| 1 | 573 (9.6%) | 480 (8.0%) | 308 (5.1%) |
| 2 | 1,775 (29.6%) | 1,477 (24.7%) | 1,307 (21.8%) |
| 3 | 2,335 (39.0%) | 2,589 (43.2%) | 2,582 (43.1%) |
| 4 | 962 (16.1%) | 1,069 (17.9%) | 1,270 (21.2%) |
| 5 | 206 (3.4%) | 265 (4.4%) | 371 (6.2%) |
| 6 | 58 (1.0%) | 60 (1.0%) | 96 (1.6%) |
| 7 | 8 (0.1%) | 11 (0.2%) | 18 (0.3%) |
| 8 | 1 (0.0%) | 2 (0.0%) | 4 (0.1%) |

Note: T1, year 2022; T2, year 2023; T3, year 2024.

# Table S4 Model fit indices and model comparisons for random intercept cross-lagged panel models

| Models | S-B*χ*^2^ | *df* | *c* | S-BΔχ^2^ | Δ*df* | *P* value | RMSEA | 95%CI | CFI | TLI | SRMR | AIC | BIC |
| --- | --- | --- | --- | --- | --- | --- | --- | --- | --- | --- | --- | --- | --- |
| Model a | 1.304 | 1 | 1.0343 | —— | —— | —— | 0.007 | 0.000, 0.036 | 1.000 | 0.999 | 0.003 | 173078.622 | 173252.757 |
| Model b | 478.470 | 25 | 1.1157 | —— | —— | —— | 0.055 | 0.051, 0.059 | 0.913 | 0.822 | 0.054 | 172850.833 | 173105.338 |
| Model c | 330.665 | 49 | 1.1012 | Ref | Ref | Ref | 0.031 | 0.028, 0.034 | 0.948 | 0.908 | 0.025 | 172646.951 | 172981.826 |
| Model d | 331.693 | 51 | 1.1323 | 6.043 | 2 | 0.049 | 0.030 | 0.027, 0.033 | 0.948 | 0.912 | 0.026 | 172654.419 | 172974.899 |
| Model e | 330.260 | 51 | 1.1025 | -0.015 | 2 | 1.000 | 0.030 | 0.027, 0.033 | 0.948 | 0.912 | 0.025 | 172642.952 | 172964.432 |
| Model f | 331.371 | 53 | 1.1338 | 7.553 | 4 | 0.109 | 0.030 | 0.027, 0.033 | 0.949 | 0.916 | 0.026 | 172650.538 | 172958.624 |

Note: *S-Bχ*^2^, Satorra-Bentler scaled chi-square; *df*, degrees of freedom; *c*, scaling correction factor for chi-square test using MLR; CFI, Comparative Fit Index; TLI, Tucker-Lewis Index; RMSEA, Root Mean Square Error of Approximation; SRMR, Standardized Root Mean Square Residual; AIC, Akaike Information Criterion; BIC, Bayesian Information Criterion; Model a, baseline model (unconstrained model); Model b, controlled for time-invariant covariates; Model c, controlled for time-invariant and time-varying covariates; Model d, building further on Model c, model with autoregressive paths fixed to be time-invariant; Model e, building further on Model c, model with cross-lagged paths fixed to be time-invariant; Model f, building further on Model c, model with autoregressive and cross-lagged paths fixed to be time-invariant; Model c was defined a reference model. Model comparisons were conducted exclusively for models c–f.

# Table S5 Non-standardized and standardized estimates for the cross-lagged and autoregressive paths of the final random intercept cross-lagged panel model (Model f)

| Paths |  | Estimate | SE | Est./SE | *P* Value |
| --- | --- | --- | --- | --- | --- |
| Within-person component |  |  |  |  |  |
| UL T1→UL T2 | Non-standardized | 0.097 | 0.020 | 4.818 | < 0.001 |
|  | Standardized | 0.097 | 0.020 | 4.853 | < 0.001 |
| UL T2→UL T3 | Non-standardized | 0.097 | 0.020 | 4.818 | < 0.001 |
|  | Standardized | 0.093 | 0.020 | 4.757 | < 0.001 |
| DS T1→DS T2 | Non-standardized | 0.248 | 0.027 | 9.098 | < 0.001 |
|  | Standardized | 0.225 | 0.025 | 8.911 | < 0.001 |
| DS T2→DS T3 | Non-standardized | 0.248 | 0.027 | 9.098 | < 0.001 |
|  | Standardized | 0.232 | 0.026 | 8.812 | < 0.001 |
| DS T1→UL T2 | Non-standardized | 0.010 | 0.002 | 3.963 | < 0.001 |
|  | Standardized | 0.065 | 0.017 | 3.919 | < 0.001 |
| DS T2→UL T3 | Non-standardized | 0.010 | 0.002 | 3.963 | < 0.001 |
|  | Standardized | 0.069 | 0.018 | 3.912 | < 0.001 |
| UL T1→DS T2 | Non-standardized | 0.081 | 0.116 | 0.699 | 0.484 |
|  | Standardized | 0.011 | 0.016 | 0.698 | 0.485 |
| UL T2→DS T3 | Non-standardized | 0.081 | 0.116 | 0.699 | 0.484 |
|  | Standardized | 0.010 | 0.015 | 0.698 | 0.485 |
| Between person component |  |  |  |  |  |
| UL⟷DS | Non-standardized | 0.752 | 0.110 | 6.808 | < 0.001 |
|  | Standardized | 0.393 | 0.049 | 8.080 | < 0.001 |

Note: DS, depressive symptoms; UL, unhealthy lifestyle; SE, standard error; Model f, building further on Model c, model with autoregressive and cross-lagged paths fixed to be time-invariant; T1, year 2022; T2, year 2023; T3, year 2024.

# Table S6 Associations of covariates with depressive symptoms and the number of unhealthy lifestyle factors in the final random intercept cross-lagged panel model (Model f)

| Paths | Non-standardized | | | |  | Standardized | | | |
| --- | --- | --- | --- | --- | --- | --- | --- | --- | --- |
|  | Estimate | SE | Est./SE | *P* Value |  | Estimate | SE | Est./SE | *P* Value |
| Age T1→UL T1 | 0.151 | 0.022 | 6.977 | < 0.001 |  | 0.143 | 0.021 | 6.974 | < 0.001 |
| Boys T1→UL T1 | -0.033 | 0.025 | -1.291 | 0.197 |  | -0.016 | 0.012 | -1.291 | 0.197 |
| Han T1→UL T1 | -0.316 | 0.106 | -2.998 | 0.003 |  | -0.033 | 0.011 | -2.955 | 0.003 |
| Living with father/mother T1→UL T1 | -0.197 | 0.084 | -2.335 | 0.020 |  | -0.030 | 0.013 | -2.325 | 0.020 |
| Urban T1→UL T1 | 0.074 | 0.026 | 2.861 | 0.004 |  | 0.035 | 0.012 | 2.861 | 0.004 |
| Living on campus T1→UL T1 | 0.069 | 0.037 | 1.869 | 0.062 |  | 0.027 | 0.014 | 1.871 | 0.061 |
| Senior high school T1→UL T1 | 0.236 | 0.067 | 3.514 | < 0.001 |  | 0.080 | 0.023 | 3.528 | < 0.001 |
| BMI T1→UL T1 | 0.011 | 0.013 | 0.821 | 0.412 |  | 0.010 | 0.013 | 0.821 | 0.411 |
|  |  |  |  |  |  |  |  |  |  |
| Age T1→DS T1 | 0.631 | 0.153 | 4.132 | < 0.001 |  | 0.084 | 0.020 | 4.128 | < 0.001 |
| Boys T1→DS T1 | -0.355 | 0.169 | -2.101 | 0.036 |  | -0.024 | 0.011 | -2.115 | 0.034 |
| Han T1→DS T1 | 0.528 | 0.798 | 0.662 | 0.508 |  | 0.008 | 0.012 | 0.661 | 0.508 |
| Living with father/mother T1→DS T1 | -2.149 | 0.602 | -3.567 | < 0.001 |  | -0.046 | 0.013 | -3.514 | < 0.001 |
| Urban T1→DS T1 | 0.378 | 0.175 | 2.163 | 0.031 |  | 0.025 | 0.012 | 2.171 | 0.030 |
| Living on campus T1→DS T1 | 0.097 | 0.240 | 0.407 | 0.684 |  | 0.005 | 0.013 | 0.407 | 0.684 |
| Senior high school T1→DS T1 | -0.314 | 0.469 | -0.669 | 0.503 |  | -0.015 | 0.022 | -0.670 | 0.503 |
| BMI T1→DS T1 | 0.016 | 0.096 | 0.163 | 0.871 |  | 0.002 | 0.013 | 0.163 | 0.871 |
|  |  |  |  |  |  |  |  |  |  |
| Senior high school T1→UL T2 | 0.420 | 0.039 | 10.854 | < 0.001 |  | 0.153 | 0.014 | 11.046 | < 0.001 |
| BMI T1→UL T2 | 0.023 | 0.013 | 1.734 | 0.083 |  | 0.022 | 0.013 | 1.736 | 0.083 |
|  |  |  |  |  |  |  |  |  |  |
| Senior high school T1→DS T2 | 1.754 | 0.282 | 6.227 | < 0.001 |  | 0.083 | 0.013 | 6.194 | < 0.001 |
| BMI T1→DS T2 | 0.079 | 0.103 | 0.773 | 0.440 |  | 0.010 | 0.013 | 0.773 | 0.440 |
|  |  |  |  |  |  |  |  |  |  |
| Senior high school T1→UL T3 | 0.314 | 0.036 | 8.799 | < 0.001 |  | 0.123 | 0.014 | 8.849 | < 0.001 |
| BMI T1→UL T3 | 0.001 | 0.014 | 0.068 | 0.945 |  | 0.001 | 0.013 | 0.068 | 0.945 |

Note: DS, depressive symptoms; UL, unhealthy lifestyle; SE, standard error; BMI, body mass index; Model f, building further on Model c, model with autoregressive and cross-lagged paths fixed to be time-invariant; T1, year 2022; T2, year 2023; T3, year 2024.

# Table S7 Standardized cross-lagged path estimates and model fit indices from sensitivity analyses using random-intercept cross-lagged panel models

| Models | Estimate | SE | *P* Value | Model fit indices |
| --- | --- | --- | --- | --- |
| Model g |  |  |  | RMSEA = 0.032  CFI = 0.946  TLI = 0.904  SRMR = 0.026 |
| DS T1→UL T2 | 0.056 | 0.024 | 0.018 |  |
| DS T2→UL T3 | 0.081 | 0.021 | < 0.001 |  |
| UL T1→DS T2 | 0.021 | 0.020 | 0.287 |  |
| UL T2→DS T3 | 0.017 | 0.019 | 0.361 |  |
| Model h |  |  |  | RMSEA = 0.031  CFI = 0.948  TLI = 0.908  SRMR =0.025 |
| DS T1→UL T2 | 0.070 | 0.022 | 0.001 |  |
| DS T2→UL T3 | 0.076 | 0.021 | < 0.001 |  |
| UL T1→DS T2 | 0.014 | 0.019 | 0.456 |  |
| UL T2→DS T3 | 0.014 | 0.019 | 0.466 |  |
| Model i |  |  |  | RMSEA = 0.032  CFI = 0.946  TLI = 0.905  SRMR = 0.026 |
| DS T1→UL T2 | 0.053 | 0.023 | 0.023 |  |
| DS T2→UL T3 | 0.079 | 0.021 | < 0.001 |  |
| UL T1→DS T2 | 0.021 | 0.020 | 0.275 |  |
| UL T2→DS T3 | 0.018 | 0.019 | 0.343 |  |
| Model j |  |  |  | RMSEA = 0.032  CFI = 0.944  TLI = 0.901  SRMR = 0.026 |
| DS T1→UL T2 | 0.079 | 0.022 | < 0.001 |  |
| DS T2→UL T3 | 0.069 | 0.022 | 0.001 |  |
| UL T1→DS T2 | 0.008 | 0.019 | 0.667 |  |
| UL T2→DS T3 | 0.024 | 0.019 | 0.200 |  |
| Model k |  |  |  | RMSEA = 0.031  CFI = 0.948  TLI = 0.908  SRMR =0.025 |
| DS T1→UL T2 | 0.070 | 0.022 | 0.001 |  |
| DS T2→UL T3 | 0.077 | 0.021 | < 0.001 |  |
| UL T1→DS T2 | 0.014 | 0.019 | 0.470 |  |
| UL T2→DS T3 | 0.014 | 0.019 | 0.460 |  |
| Model l |  |  |  | RMSEA = 0.032  CFI = 0.944  TLI = 0.901  SRMR = 0.025 |
| DS T1→UL T2 | 0.077 | 0.022 | < 0.001 |  |
| DS T2→UL T3 | 0.068 | 0.022 | 0.002 |  |
| UL T1→DS T2 | 0.007 | 0.019 | 0.695 |  |
| UL T2→DS T3 | 0.025 | 0.019 | 0.177 |  |
| Model m |  |  |  | RMSEA = 0.031  CFI = 0.948  TLI = 0.908  SRMR = 0.025 |
| DS T1→UL T2 | 0.073 | 0.022 | 0.001 |  |
| DS T2→UL T3 | 0.078 | 0.021 | < 0.001 |  |
| UL T1→DS T2 | 0.016 | 0.019 | 0.397 |  |
| UL T2→DS T3 | 0.010 | 0.018 | 0.587 |  |

Note: DS, depressive symptoms; UL, unhealthy lifestyle; SE, standard error; CFI, Comparative Fit Index; TLI, Tucker-Lewis Index; RMSEA, Root Mean Square Error of Approximation; SRMR, Standardized Root Mean Square Residual; Model g excluded inadequate sleep from the number of unhealthy lifestyle factors; Model h excluded the sleep-related item from the depressive symptom score; Model i excluded sleep-related components from both the number of unhealthy lifestyles and the depressive symptom score; Model j excluded physical inactivity from the number of unhealthy lifestyles; Model k excluded physical inactivity-related items from the depressive symptom score; Model l: excluded physical inactivity related components from both the number of unhealthy lifestyles and the depressive symptom score; Model m was conducted including all 6,165 participants with incomplete data; Models g-m were all adjusted for both time-invariant and time-varying covariates; T1, year 2022; T2, year 2023; T3, year 2024.

# Table S8 Latent class linear mixed model selection

| Trajectory (n) | APPA | | | | OCC | | | | Participants (%) | | | | BIC | Log likelihood | Relative entropy |
| --- | --- | --- | --- | --- | --- | --- | --- | --- | --- | --- | --- | --- | --- | --- | --- |
|  | Traj. 1 | Traj. 2 | Traj. 3 | Traj. 4 | Traj. 1 | Traj. 2 | Traj. 3 | Traj. 4 | Traj. 1 | Traj. 2 | Traj. 3 | Traj. 4 |  |  |  |
| 2 | 0.951 | 0.887 | —— | —— | 2.661 | 57.239 | —— | —— | 0.880 | 0.120 | —— | —— | 120164 | -60029.6 | 0.928 |
| 3 | 0.874 | 0.765 | 0.878 | —— | 2.501 | 12.151 | 126.2 | —— | 0.735 | 0.211 | 0.054 | —— | 119618 | -59734.9 | 0.885 |
| 4 | NA | 0.591 | 0.626 | 0.821 | NA | 0.456 | 6.603 | 116.74 | 0.760 | 0.202 | 0.038 | NA | 120958 | -60383.3 | 0.787 |

Note: APPA, Average Posterior Probability of Assignment; OCC, Odds of Correct Classification; BIC, Bayesian Information Criterion.

# Table S9 Detailed descriptions of three depressive symptom trajectories

| Depressive symptom trajectories | Description |
| --- | --- |
| Low (n = 4,401) | - Throughout the entire age range, the “low” trajectory maintained consistently low levels of depressive symptoms. - Although depressive symptoms showed a gradual increase with age (mean_age=11_ vs. mean_age=18_ = 4.43 vs. 7.16; p < 0.001), the absolute symptom levels remained well below those observed in the other two trajectories. |
| Fluctuating (n = 1,264) | - At age 11, the “fluctuating” trajectory exhibited similar levels of depressive symptoms as the “high peaking” trajectory (mean comparisons: 14.88 vs. 15.86; p = 0.157). - As age increased, the “fluctuating” trajectory showed the opposite pattern: initial decline followed by an increase. - Between ages 13 and 16, depressive symptoms increased steadily, indicating a transition from a relatively lower symptom level to a higher-risk phase (mean_age=13_ vs. mean_age=16_ = 12.64 vs. 17.42; p < 0.001). - After age 16, the depressive symptoms of the “fluctuating” trajectory reached a level comparable to the “high peaking” trajectory and continued to rise further. |
| High peaking (n = 323) | - At age 11, the “high peaking” trajectory exhibited similar levels of depressive symptoms as “fluctuating” trajectory (mean comparisons: 15.86 vs. 14.88; p = 0.157). - As age increased, the “high peaking” trajectory showed a pattern of initial increase followed by a decline in depressive symptoms. - Depressive symptoms reached their highest levels during early to mid-adolescence, peaking around ages 13–14 (mean_age=11_ vs. mean_age=14_ = 15.86 vs. 28.25; p < 0.001). - Following the peak, symptom levels declined progressively with increasing age, particularly after age 15 (mean_age=15_ vs. mean_age=18_ = 23.79 vs. 14.00; p = 0.003). - Despite this downward trend, depressive symptoms in the “high peaking” trajectory remained higher than those in the “low” trajectory across most of adolescence, indicating persistent vulnerability (mean_age=18_ comparisons: 14.00 vs. 7.16; p = 0.007). |

# Table S10 Differences in demographic variables and unhealthy lifestyle factors at the three waves across depressive symptom trajectories

| Variables | T1 | | | | | T2 | | | | | T3 | | | | |
| --- | --- | --- | --- | --- | --- | --- | --- | --- | --- | --- | --- | --- | --- | --- | --- |
|  | Depressive symptoms trajectory classes [mean±SD/n (%)] | | | *χ*^2^/*F* | *P* value | Depressive symptoms trajectory classes [mean±SD/n (%)] | | | *χ*^2^/*F* | *P* value | Depressive symptoms trajectory classes [mean±SD/n (%)] | | | *χ*^2^/*F* | *P* value |
|  | Low | Fluctuating | High-peaking |  |  | Low | Fluctuating | High-peaking |  |  | Low | Fluctuating | High-peaking |  |  |
| Age | 12.73±1.42 | 12.79±1.35 | 12.85±1.37 | 1.92 | 0.147 | 13.47±1.36 | 13.54±1.29 | 13.58±1.30 | 2.12 | 0.120 | 14.47±1.36 | 14.53±1.29 | 14.59±1.31 | 2.04 | 0.131 |
| Gender |  |  |  | 25.22 | <0.001 |  |  |  | 25.22 | <0.001 |  |  |  | 25.22 | <0.001 |
| Boys | 2,267 (51.5) | 636 (49.5) | 120 (37.2) |  |  | 2,267 (51.5) | 636 (49.5) | 120 (37.2) |  |  | 2,267 (51.5) | 636 (49.5) | 120 (37.2) |  |  |
| Girls | 2,134 (48.5) | 638 (50.5) | 203 (62.8) |  |  | 2,134 (48.5) | 638 (50.5) | 203 (62.8) |  |  | 2,134 (48.5) | 638 (50.5) | 203 (62.8) |  |  |
| Ethnicity |  |  |  | 0.11 | 0.949 |  |  |  | 0.104 | 0.949 |  |  |  | 0.104 | 0.949 |
| Han | 4,350 (98.8) | 1,248 (98.7) | 319 (98.8) |  |  | 4,350 (98.8) | 1,248 (98.7) | 319 (98.8) |  |  | 4,350 (98.8) | 1,248 (98.7) | 319 (98.8) |  |  |
| Other | 51 (1.2) | 16 (1.3) | 4 (1.2) |  |  | 51 (1.2) | 16 (1.3) | 4 (1.2) |  |  | 51 (1.2) | 16 (1.3) | 4 (1.2) |  |  |
| Region |  |  |  | 12.17 | 0.002 |  |  |  | 11.55 | 0.003 |  |  |  | 11.49 | 0.003 |
| Urban | 2,102 (47.8) | 579 (45.8) | 183 (56.7) |  |  | 2,103 (47.8) | 583 (46.1) | 183 (56.7) |  |  | 2,109 (47.9) | 583 (46.1) | 183 (56.7) |  |  |
| Suburban | 2,299 (52.2) | 685 (54.2) | 140 (43.3) |  |  | 2,298 (52.2) | 681 (53.9) | 140 (43.4) |  |  | 2,292 (52.1) | 681 (53.9) | 140 (43.3) |  |  |
| Living with father/mother |  |  |  | 26.54 | <0.001 |  |  |  | 17.89 | <0.001 |  |  |  | 22.82 | <0.001 |
| Yes | 4,310 (97.9) | 1,218 (96.4) | 303 (93.8) |  |  | 4,297 (97.6) | 1,223 (96.8) | 303 (93.8) |  |  | 4,331 (98.4) | 1,224 (96.8) | 308 (95.4) |  |  |
| No | 91 (2.1) | 46 (3.6) | 20 (6.2) |  |  | 104 (2.4) | 41 (3.2) | 20 (6.2) |  |  | 70 (1.6) | 40 (3.2) | 15 (4.6) |  |  |
| Living on campus |  |  |  | 4.30 | 0.116 |  |  |  | 6.57 | 0.038 |  |  |  | 7.99 | 0.018 |
| Yes | 938 (21.3) | 297 (23.5) | 80 (24.8) |  |  | 880 (20.0) | 288 (22.8) | 77 (23.8) |  |  | 869 (19.7) | 278 (22.0) | 82 (25.4) |  |  |
| No | 3,463 (78.7) | 967 (76.5) | 243 (75.2) |  |  | 3,521 (80.0) | 976 (77.2) | 246 (76.2) |  |  | 3,532 (80.3) | 986 (78.0) | 241 (74.6) |  |  |
| Education level |  |  |  | 0.24 | 0.889 |  |  |  | 0.36 | 0.836 |  |  |  | 0.28 | 0.871 |
| Junior high school | 3,745 (85.1) | 1,078 (85.3) | 272 (84.2) |  |  | 3,645 (82.8) | 1,038 (82.1) | 266 (82.4) |  |  | 3,396 (77.2) | 969 (76.7) | 246 (76.2) |  |  |
| Senior high school | 656 (14.9) | 186 (14.7) | 51 (15.8) |  |  | 756 (17.2) | 226 (17.9) | 57 (17.6) |  |  | 1,005 (22.8) | 295 (23.3) | 77 (23.8) |  |  |
| BMI | 21.54±4.45 | 21.67±4.60 | 21.27±4.04 | 1.08 | 0.340 | 21.68±4.63 | 21.91±4.80 | 21.64±4.32 | 1.34 | 0.262 | 22.10±4.72 | 22.15±4.85 | 21.96±4.55 | 0.21 | 0.814 |
| Smoking |  |  |  | 73.50 | <0.001 |  |  |  | 51.95 | <0.001 |  |  |  | 58.43 | < 0.001 |
| Yes | 31 (0.7) | 39 (3.1) | 17 (5.3) |  |  | 36 (0.8) | 40 (3.2) | 13 (4.0) |  |  | 54 (1.2) | 45 (3.6) | 20 (6.2) |  |  |
| No | 4,370 (99.3) | 1,225 (96.9) | 306 (94.7) |  |  | 4,365 (99.2) | 1,224 (96.8) | 310 (96.0) |  |  | 4,347 (98.8) | 1,219 (96.4) | 303 (93.8) |  |  |
| Alcohol consumption |  |  |  | 135.62 | <0.001 |  |  |  | 153.06 | <0.001 |  |  |  | 107.79 | < 0.001 |
| Yes | 173 (3.9) | 131 (10.4) | 52 (16.1) |  |  | 186 (4.2) | 126 (10.0) | 62 (19.2) |  |  | 263 (6.0) | 145 (11.5) | 64 (19.8) |  |  |
| No | 4,228 (96.1) | 1,133 (89.6) | 271 (83.9) |  |  | 4,215 (95.8) | 1,138 (90.0) | 261 (80.8) |  |  | 4,138 (94.0) | 1,119 (88.5) | 259 (80.2) |  |  |
| Physical inactivity |  |  |  | 21.56 | <0.001 |  |  |  | 16.60 | <0.001 |  |  |  | 24.89 | < 0.001 |
| Yes | 3,761 (85.5) | 1,144 (90.5) | 280 (86.7) |  |  | 3,764 (85.5) | 1,137 (90.0) | 277 (85.8) |  |  | 3,717 (84.5) | 1,138 (90.0) | 276 (85.4) |  |  |
| No | 640 (14.5) | 120 (9.5) | 43 (13.3) |  |  | 637 (14.5) | 127 (10.0) | 46 (14.2) |  |  | 684 (15.5) | 126 (10.0) | 47 (14.6) |  |  |
| Low fruit/vegetable intake |  |  |  | 87.43 | <0.001 |  |  |  | 138.78 | <0.001 |  |  |  | 117.53 | < 0.001 |
| Yes | 1,075 (24.4) | 456 (36.1) | 125 (38.7) |  |  | 1,078 (24.5) | 516 (40.8) | 118 (36.5) |  |  | 1,214 (27.6) | 545 (43.1) | 124 (38.4) |  |  |
| No | 3,326 (75.6) | 808 (63.9) | 198 (61.3) |  |  | 3,323 (75.5) | 748 (59.2) | 205 (63.5) |  |  | 3,187 (72.4) | 719 (56.9) | 199 (61.6) |  |  |
| Unhealthy diet |  |  |  | 51.44 | <0.001 |  |  |  | 110.15 | <0.001 |  |  |  | 61.05 | < 0.001 |
| Yes | 226 (5.1) | 116 (9.2) | 42 (13.0) |  |  | 232 (5.3) | 165 (13.1) | 46 (14.2) |  |  | 290 (6.6) | 156 (12.3) | 47 (14.6) |  |  |
| No | 4,175 (94.9) | 1,148 (90.8) | 281 (87.0) |  |  | 4,169 (94.7) | 1,099 (86.9) | 277 (85.8) |  |  | 4,111 (93.4) | 1,108 (87.7) | 276 (85.4) |  |  |
| Excessive screen time |  |  |  | 30.48 | <0.001 |  |  |  | 77.48 | <0.001 |  |  |  | 109.03 | < 0.001 |
| Yes | 238 (5.4) | 112 (8.9) | 35 (10.8) |  |  | 285 (6.5) | 152 (12.0) | 56 (17.3) |  |  | 407 (9.2) | 207 (16.4) | 81 (25.1) |  |  |
| No | 4,163 (94.6) | 1,152 (91.1) | 288 (89.2) |  |  | 4,116 (93.5) | 1,112 (88.0) | 267 (82.7) |  |  | 3,994 (90.8) | 1,057 (83.6) | 242 (74.9) |  |  |
| Sedentary behavior |  |  |  | 16.97 | <0.001 |  |  |  | 3.25 | 0.197 |  |  |  | 2.69 | 0.26 |
| Yes | 3,282 (74.6) | 871 (68.9) | 229 (70.9) |  |  | 3,434 (78.0) | 957 (75.7) | 254 (78.6) |  |  | 3,635 (82.6) | 1,023 (80.9) | 272 (84.2) |  |  |
| No | 1,119 (25.4) | 393 (31.1) | 94 (29.1) |  |  | 967 (22.0) | 307 (24.3) | 69 (21.4) |  |  | 766 (17.4) | 241 (19.1) | 51 (15.8) |  |  |
| Inadequate sleep |  |  |  | 30.61 | <0.001 |  |  |  | 60.78 | <0.001 |  |  |  | 25.52 | < 0.001 |
| Yes | 2,851 (64.8) | 880 (69.6) | 252 (78.0) |  |  | 3,058 (69.5) | 997 (78.9) | 266 (82.4) |  |  | 3,319 (75.4) | 1,035 (81.9) | 260 (80.5) |  |  |
| No | 1,550 (35.2) | 384 (30.4) | 71 (22.0) |  |  | 1,343 (30.5) | 267 (21.1) | 57 (17.6) |  |  | 1,082 (24.6) | 229 (18.1) | 63 (19.5) |  |  |
| Numbers of unhealthy lifestyle factors | 2.64±1.02 | 2.97±1.11 | 3.20±1.25 | 78.06 | <0.001 | 2.74±0.98 | 3.24±1.10 | 3.38±1.20 | 156.04 | <0.001 | 2.93±1.01 | 3.40±1.11 | 3.54±1.24 | 134.04 | < 0.001 |

Note: SD, standard deviation; BMI, body mass index; T1, year 2022; T2, year 2023; T3, year 2024.

# Table S11 Sensitivity analyses of the association between depressive symptom trajectories, depressive symptoms at T1, and the number of unhealthy lifestyle factors at T3

|  | Model S1-GLMM (Poisson) | | Model S2-LMM | | Model S3-GLMM (logit) | |
| --- | --- | --- | --- | --- | --- | --- |
|  | AIRR (95%CI) | *P* value | β (95%CI) | *P* value | AOR (95%CI) | *P* value |
| Baseline CESD | 1.025 (1.010, 1.041) | 0.001 | —— | —— | —— | —— |
| Depressive symptoms  trajectory classes |  |  |  |  |  |  |
| Traj. 2 : Traj. 1 | —— | —— | 0.371 (0.308, 0.433) | < 0.001 | 2.156 (1.868, 2.489) | < 0.001 |
| Traj. 3 : Traj. 1 | —— | —— | 0.481 (0.369, 0.593) | < 0.001 | 2.516 (1.957, 3.235) | < 0.001 |
| Traj. 3 : Traj. 2 | —— | —— | 0.110 (-0.009, 0.230) | 0.070 | 1.167 (0.894, 1.522) | 0.256 |

Note: AIRR, adjusted incidence rate ratios; AOR, adjusted odds ratio; CESD, the Center for Epidemiologic Studies Depression Scale; Traj. 1, Low; Traj. 2, Fluctuating; Traj. 3, High-peaking; Model S1-GLMM, Poisson generalized linear mixed model; Model S2-LMM, linear mixed-effects models; Model S3-GLMM, generalized linear mixed model with logit, GLMM - binomial distribution, logit link (canonical); Controlling for age, gender, ethnicity, region, education level, BMI, living with father/mother, living on campus, and the number of unhealthy lifestyle factors in T1.

# Table S12 Associations of the number of unhealthy lifestyle factors at T1 with depressive symptoms at T3 and depressive symptom trajectories

| Independent variables | Dependent variables | AIRR/AOR (95%CI) | *P* value |
| --- | --- | --- | --- |
| Numbers of unhealthy lifestyle factors at T1 | Depressive symptoms in T3 | 1.015 (0.995, 1.036) | 0.152 |
| Numbers of unhealthy lifestyle factors at T1 | Traj. 2: Traj. 1 | 1.055 (0.975, 1.142) | 0.183 |
| Numbers of unhealthy lifestyle factors at T1 | Traj. 3: Traj. 1 | 1.121 (0.950, 1.323) | 0.175 |
| Numbers of unhealthy lifestyle factors at T1 | Traj. 3: Traj. 2 | 0.998 (0.880, 1.132) | 0.978 |

Note: AIRR, adjusted incidence rate ratio; Traj. 1, Low; Traj. 2, Fluctuating; Traj. 3, High-peaking; Controlling for age, gender, ethnicity, region, education level, BMI, living with father/mother, living on campus, and depressive symptoms in T1.
